# Effect of Peer Mentors in Diabetes Self-management vs Usual Care on Outcomes in US Veterans With Type 2 Diabetes: A Randomized Clinical Trial

**DOI:** 10.1001/jamanetworkopen.2020.16369

**Published:** 2020-09-11

**Authors:** Judith A. Long, Valerie S. Ganetsky, Anne Canamucio, Tanisha N. Dicks, Michele Heisler, Steven C. Marcus

**Affiliations:** 1 Corporal Michael J. Crescenz VA Medical Center, VA Center for Health Equity Research and Promotion, Philadelphia, Pennsylvania; 2 Division of General Internal Medicine, Perelman School of Medicine, University of Pennsylvania, Philadelphia; 3 Division of Addiction Medicine and Urban Health Institute, Cooper University Hospital, Camden, New Jersey; 4 Veterans Integrated Service Network 4, Center for Evaluation of PACT, Philadelphia, Pennsylvania; 5 Department of Internal Medicine, University of Michigan, Ann Arbor; 6 Center for Clinical Management Research, Ann Arbor Veterans’ Affairs Healthcare System, Ann Arbor, Michigan; 7 Department of Health Behavior and Health Education, School of Public Health, University of Michigan, Ann Arbor; 8 Michigan Center for Diabetes Translation Research, University of Michigan, Ann Arbor; 9 School of Social Policy and Practice, University of Pennsylvania, Philadelphia

## Abstract

**Importance:**

Diabetes is a substantial public health issue. Peer mentoring is a low-cost intervention for improving glycemic control in patients with diabetes. However, long-term effects of peer mentoring and creation of sustainable models are not well studied.

**Objective:**

Assess the effects of a peer support intervention for improving glycemic control in patients with diabetes and evaluate a model in which former mentees serve as mentors.

**Design, Setting, and Participants:**

A randomized clinical trial was conducted from September 27, 2012, to March 21, 2018, at the Corporal Michael J. Crescenz Medical Center. US veterans with type 2 diabetes aged 30 to 75 years with hemoglobin A_1C_ (HbA_1c_) greater than 8% received support over 6 months from peers with prior poor glycemic control but who had achieved HbA_1c_ less than or equal to 7.5% (phase 1). Phase 1 mentees were then randomized to become a mentor or not to new randomly assigned participants in phase 2. Outcomes were assessed at 6 and 12 months. Data were analyzed from October 5, 2016, to September 4, 2018.

**Interventions:**

Mentors who received an initial training session and monthly reinforcement training were assigned 1 mentee and given $20 for each month they contacted their mentee at least weekly.

**Main Outcomes and Measures:**

Primary outcome was HbA_1c_ change at 6 months. Secondary outcomes included HbA_1c_ change at 12 months and change in low-density lipoprotein, blood pressure, diabetes quality of life, and depression symptoms at 6 and 12 months.

**Results:**

The study enrolled 365 participants into phase 1 and 122 participants into phase 2. Most participants were Black (341 [66%]) and male (454 [96%]), with a mean (SD) age of 60 (7.5) years. Mean phase 1 HbA_1c_ change at 6 months for usual care was −0.20% (95% CI, −0.46% to 0.06%) vs −0.52% (95% CI, −0.76% to −0.29%) for mentees (*P* = .06). Mean phase 2 HbA_1c_ change at 6 months for usual care was −0.46% (95% CI, −1.02% to 0.10%) vs 0.08% (95% CI, −0.42% to 0.57%) for mentees (*P* = .16). There were no differences in secondary outcomes or HbA_1c_ levels at 12 months. There was no benefit to past mentees who became mentors.

**Conclusions and Relevance:**

In this randomized clinical trial, a peer mentor intervention did not improve 6-month HbA_1c_ levels and did not have sustained benefits.

**Trial Registration:**

ClinicalTrials.gov Identifier: NCT01651117

## Key Points

**Question:**

What are the effects of a peer support intervention in participants with poorly controlled type 2 diabetes?

**Findings:**

In phase 1 of this 2-phase randomized clinical trial involving 365 participants, a peer mentoring intervention did not improve hemoglobin A_1C_ levels at 6 months and did not improve outcomes at 12 months. In phase 2 with 122 participants, receiving mentoring from a past mentee did not improve glycemic control and may have worsened mentees’ control.

**Meaning:**

Future studies may be needed to determine optimal practices to create long-term, sustainable peer-mentoring models.

## Teaser

This randomized clinical trial assesses the effects of a peer support intervention for improving glycemic control vs usual care on hemoglobin A_1c_ levels in US veterans with diabetes.

## Introduction

Diabetes remains a major public health issue, affecting an estimated 9.4% of the US population.^1^ The prevalence of diabetes among veterans is higher at approximately 16%.^2^ Care for patients with diabetes represents a substantial portion of the use of Department of Veterans Affairs (VA) resources.^3,4^ Achieving good glycemic and other risk factor control through lifestyle changes and medication adherence requires substantial patient engagement in self-care.^5,6,7,8^ Yet, patients face many barriers to effective self-care.^9,10,11,12^

Diabetes self-care activities take place primarily outside of clinical encounters. Intensive clinic-based programs have been reported to be effective in improving self-care behaviors; however, they are often resource intensive, and participant engagement wanes over time.^13,14^ Peer support models that include peers with the same chronic illness and experiential knowledge may help augment patients’ existing social support structures and improve self-care.^15,16,17,18,19,20,21^ Models using peers, such as shared medical appointments and community health worker programs, have been shown to improve diabetes clinical outcomes.^15,16,17,18,19,20^

A more informal, flexible, and potentially inexpensive means of providing peer support is through volunteer peer coaches or mentors. Peer mentor programs have been shown to improve glycemic control and adherence to medications, diet, exercise, and blood glucose monitoring.^22,23,24,25,26^ The success of peer mentor programs is thought to be due in part to the reciprocity created through sharing of similar life experiences.^21^ Peer support may actually be just as beneficial to mentors as it is to mentees.^25,26,27^ A study by Heisler et al^24^ found that, compared with receiving nurse care management, veterans in a reciprocal peer support program experienced improved diabetes control. However, mentors in an obesity study experienced weight regain, and mentors’ weight changes were not associated with mentees’ success.^28^

A previous study^23^ showed the benefits of a telephone-based peer mentor model in which mentors with previously poorly controlled diabetes but with good control became a mentor; however, it is unclear if there are long-term benefits of this model. Given this lack of evidence regarding the long-term impact of peer mentoring, the limited evidence examining reciprocal peer support models, and inconclusive evidence about the clinical benefits to mentors in peer support models, this study builds on prior literature of telephone-based peer support to explore a potentially sustainable model in which former mentees serve as mentors.^22,23,24^ Our main hypotheses were (1) patients with diabetes and poor glycemic control would benefit from peer mentoring from peers with previously poor glycemic control but who had achieved good control, (2) patients with poor glycemic control would benefit from peer mentoring from former mentees who became mentors, and (3) becoming a mentor would provide additional benefit to those who had previously been mentees.

## Methods

We conducted a randomized clinical trial in 2 phases. In phase 1, patients with diabetes and poor glycemic control were randomized to receive mentoring from peers with well-controlled diabetes whose diabetes was once in poor control or to usual care (phase 1 mentees vs usual care). In phase 2, different patients with poor glycemic control were randomized to receive mentoring from former mentees in phase 1 or to usual care (phase 2 mentoring from former mentee vs usual care). To assess whether becoming a mentor in phase 2 was associated with any benefit for phase 1 mentees, those patients were randomized to either become a mentor or not in phase 2 (phase 2 mentors vs nonmentors). A qualitative study examining the mentor-mentee relationship in-depth to explore factors associated with broader program implementation was also conducted.^29^ The study was conducted at the Corporal Michael J. Crescenz VA Medical Center. All aspects of the study were approved by the Corporal Michael J. Crescenz VA Medical Center Institutional Review Board. Enrollment began on September 27, 2012, to March 21, 2018, and follow-up was completed by October 2018. Data were analyzed from October 5, 2016, to September 4, 2018. The trial protocol is available in Supplement 1. This study followed the Consolidated Standards of Reporting Trials (CONSORT) reporting guideline for randomized clinical trials.

### Participants

Participants were identified through the electronic medical records. Patients with a diagnosis of diabetes were eligible to be a phase 1 or 2 mentee if they received their primary care from a Philadelphia or Camden VA facility and had a hemoglobin A1c (HbA_1c_) level greater than 8% on at least 2 occasions in the 24 months prior to enrollment, 1 of which was within the 3 months prior to enrollment. The HbA_1c_ is a measure of the % of hemoglobin which is glycated. An HbA_1c_ greater than 8% is considered poor control.^30^ We did not distinguish where the HbA_1c_ was drawn. Potentially eligible participants were sent a letter notifying them about the study, followed within 2 weeks by a telephone call. Phase 1 mentors had to have at least 1 HbA_1c_ less than or equal to 7.5% in the 3 months prior to enrollment but have at least 1 HbA_1c_ greater than 8% in the 3 years prior to enrollment. Former mentees who became mentors in phase 2 were not required to have achieved HbA_1c_ levels less than or equal to 7.5% to become mentors. Additional inclusion criteria included age 30-75 years, type 2 diabetes, access to a telephone for contact with mentor or mentee, and ability to understand English. Veterans (mentees and mentors) who agreed to participate completed a written consent form during their first in-person visit. All participants received $50 for each visit requiring a blood draw and survey (baseline, 6 months, and 12 months).

### Randomization and Intervention

Randomization was performed using permuted blocks with varying block size using SAS Proc Plan (SAS Institute Inc) generated by the study statistician (A.C.). We produced 3 randomization lists: (1) phase 1 veterans with poor glycemic control who received mentoring from a mentor who was once in poor control and now in good control vs usual care, (2) phase 2 veterans with poor glycemic control who received mentoring from a former mentee from phase 1 vs usual care, and (3) phase 2 former mentees who became mentors vs nonmentors. For the first 6 months, potential participants were only enrolled into phase 1. After that, participants were enrolled into phase 2 if there was a former mentee who had been randomized to be a mentor waiting to be matched. Study participants and staff were not blinded to group assignment. However, both participants and staff learned of the group assignment only after randomization, which occurred after completing the consent and baseline survey. Study investigators and analysts were blinded until follow-up analyses were performed.

Peer mentors participated in a 1-hour, 1-on-1 training session consisting of (1) instruction designed to help learn the mentee’s story, understand their motivations, help set a realistic plan for goal achievement, assess and support progress, and deal with failure in an accepting manner; (2) role-playing exercises; and (3) review of sample questions for potential mentee encounters. Each peer mentor was then matched with a mentee based on age (plus or minus 10 years), self-reported race/ethnicity, sex, and insulin use (with or without experience with insulin) and introduced to their mentees by research staff via telephone. If an appropriate mentor was not found within 2 weeks, matching criteria were loosened except for experience with insulin. Mentors were given $20 for each month they contacted or attempted to contact their mentee via telephone at least weekly. Research staff contacted mentors once per month to provide training reinforcement and ask about interactions. Mentees who became mentors in phase 2 received the same training, support, and incentive.

### Outcomes and Follow-up

The prespecified primary outcome was change in HbA_1c_ level from baseline to 6 months. Prespecified secondary outcomes included change in HbA_1c_ level from baseline to 12 months and change from baseline to 6 and 12 months in direct low-density lipoprotein (LDL), systolic blood pressure (BP), diabetes quality of life (measured by the Diabetes Distress Scale; respondents rated on a 5-point scale [with 1 indicating no distress and 5 indicating serious distress] the degree to which the following caused distress: [1] feeling overwhelmed by the demands of living with diabetes and [2] feeling that I am failing with my diabetes regimen; scores were the average of the 2 items^31^), and depression symptoms (measured by the Patient Health Questionnaire-2 scale; respondents rated on a 4-point scale [with 0 indicating not at all and 3 indicating nearly every day] the degree to which they had the following symptoms: [1] little interest or pleasure in doing things and [2] feeling down, depressed, or hopeless; scores were the sum of the 2 items^32^).^33^ All participants had a baseline visit during which BP, weight, and height were measured and surveys were administered. On the same day, participants underwent bloodwork for the collection of baseline HbA_1c_ level and LDL level. In-person data were collected at baseline, 6 months, and 12 months.

Adverse events (AEs) were reported to the Data Safety Monitoring Board biannually. Deaths and events requiring hospitalization were considered serious adverse events (SAEs). Any event that required an ambulance, outpatient surgery, or emergency department visit was considered an AE. Minor and major hypoglycemic events were also collected. Data Safety Monitoring Board members were blinded to study arm.

### Statistical Analysis

Our prespecified basic model for all analyses was an analysis of covariance comparing change in outcome (HbA_1c_ level) from baseline to 6 months by treatment group, adjusting for baseline HbA_1c_ level. To also assess change from baseline to 12 months, we used a mixed-effects model and included a time fixed effect and a patient random effect to account for correlation between time periods.

Preliminary complete-case analysis was accompanied by evaluation of complete cases compared with those missing 6-month HbA_1c_ follow-up. Every attempt was made to avoid missing outcomes, including abstracting HbA_1c_ measures from the electronic medical records when patients missed follow-up visits if it was within 30 days. To perform intent-to-treat analysis, we used multiple imputation using Markov chain Monte Carlo methods with 25 iterations and achieved a relative efficiency of more than 99%. Analyses were conducted on each iteration, and results were combined using the Rubin formula.^34^

Our final primary intent-to-treat analyses were conducted with multiple imputation by using analysis of covariance, adjusting for baseline and including a time fixed effect and a patient random effect. Additional analyses, not prespecified, were also performed. We used logistic regression to evaluate the outcome HbA_1c_ improvement greater than or equal to 1% (yes/no). We selected a 1% cutoff because this marker is a clinically meaningful decrease associated with an approximately 40% decrease in diabetic microvascular complications.^35^ We performed a nonprespecified exploratory subset analysis including only those with a baseline HbA_1c_ level greater than 8% (baseline was assessed after enrollment, and some patients who were recruited as having poorly controlled diabetes based on the electronic medical records showed good control at baseline). We also compared change in HbA_1c_ level in phase 2 by whether the mentor had improved their control in phase 1. Analyses were conducted using SAS, version 9.4 (SAS Institute Inc).

To achieve 80% power to detect a 0.8% change in HbA_1c_ level (with a standard error of 1.6) between phase 2 mentors and nonmentors, a sample of 64 patients per arm was required. To protect against attrition, we inflated that number by 10% to arrive at 72 participants per arm. Working backward to determine how many patients with poor glycemic control would be needed in phase 1, we started with 144 (72 for each arm of phase 2) and inflated again by 10% to arrive at 160 (to allow for attrition between phase 1 and 2). We thus intended to recruit 320 patients with poor control and randomize 1:1 to obtain 160 mentees and 160 usual care patients. However, mid-study evaluation revealed that attrition between phase 1 and phase 2 was higher than expected. As a result, we recruited additional patients with poor glycemic control to phase 1 to have sufficient phase 2 mentors. This process led to resources being wasted on an overly large usual care group in phase 1, and randomization was changed to 2:1. For the same reasons, we decided it was not necessary to maintain a 1:1 randomization in phase 2 and changed the enrollment target to 56 for usual care. Power for 72 vs 56 was 79.5%. All methodological changes were approved by the institutional review board.

## Results

Most participants were Black (341 [66%]) and male (454 [96%]), with a mean (SD) age of 60 (7.5) years (Table 1 and Table 2). We assessed 4501 patients for eligibility; 2524 were unable to be contacted or had other reasons to decline, 1131 patients contacted were eligible, 644 declined to participate, and 487 eligible participants were enrolled (Figure): 365 patients into phase 1 and 122 patients into phase 2. Of those who were mentees in phase 1, 142 were randomized to the phase 2 mentor vs nonmentor group (Figure). For the primary outcomes evaluating change in HbA_1c_ level at 6 months, we had follow-up HbA_1c_ data on more than 87% of participants. We imputed the following data: phase 1, 30 of 202 in the mentee arm and 16 of 154 in the usual care arm; phase 2, 10 of 68 mentoring from a former mentee arm and 3 of 47 in the usual care arm. For the comparison of former mentees randomized to become mentors and nonmentors, we imputed 9 of 70 and 11 of 69, respectively.

**Table 1.** Baseline Characteristics for Phase 1 Participants^a^

Variable	No. (%)	
	Usual care (n = 154)	Mentee (n = 202)
Age, mean (SD), y	60.6 (7.4)	59.6 (7.9)
Male	146 (94.8)	195 (96.5)
Race/ethnicity, self-reported		
White	40 (26.0)	50 (24.8)
Black	93 (60.4)	136 (67.3)
Other^b^	21 (13.6)	16 (7.9)
Hispanic	11 (7.1)	11(5.5)
Education		
<High school	6 (3.9)	12 (5.9)
High school	46 (29.9)	65 (32.2)
Some college	77 (50.0)	93 (46.0)
≥College	25 (16.2)	32 (15.9)
Partnered	78 (50.7)	95 (47.3)
Lives alone	39 (25.5)	68 (33.7)
Income, tertile		
Low (<$15 000/y)	35 (22.7)	51 (25.3)
Mid ($15 000 to <$40 000/y)	48 (31.2)	55 (27.2)
High (>$40 000/y)	43 (27.9)	67 (33.2)
Unreported (do not know or refused)	28 (18.2)	29 (14.4)
BMI, mean (SD)	32.7 (6.7)	33.1 (6.6)
General health history, mean (SD		
Self-rated health^c^	3.2 (0.9)	3.3 (0.9)
Duration of diabetes, mean (SD), y	14.2 (8.0)	13.8 (9.1)
Antihyperglycemic medications		
Oral medications only	32 (20.8)	52 (25.7)
Insulin only	48 (31.2)	50 (24.8)
Insulin + oral medications	73 (47.4)	100 (49.5)
Clinical measures, mean (SD)		
HbA_1c_	9.8 (1.6)	9.3 (1.6)
LDL	99.5 (39.0)	92.3 (32.1)
SBP	135.5 (18.4)	138.8 (18.3)
DBP	79.1 (13.4)	80.6 (13.3)
DDS2, mean (SD)^d^	2.4 (1.1)	2.5 (1.2)
PHQ-2, mean (SD)^e^	1.3 (1.6)	1.6 (1.8)

Abbreviations: BMI, body mass index (calculated as weight in kilograms divided by height in meters squared); DBP, diastolic blood pressure; DDS, Diabetes Distress Scale; HbA_1c_, hemoglobin A_1C_; LDL, low-density lipoprotein; PHQ, Patient Health Questionnaire; SBP, systolic blood pressure.

SI conversion factors: To convert LDL to mmol/L, multiply by 0.0259; HbA_1c_ to proportion of total hemoglobin, multiply by 0.01.

^a^ The number in each group represents the number of participants analyzed.

^b^ Other is a convenience category that includes multiple races (checked more than 1 box), Asian, Native Hawaiian or Pacific Islander, Alaskan Native or American Indian, other (could be written in if desired but coded as other), and missing.

^c^ Self-rated health was assessed through the Short Form Health Survey, question 1 (SF-1): “In general, would you say your health is Excellent, Very Good, Good, Fair, Poor.” Scores range from 1 indicating excellent to 5 indicating poor.

^d^ Diabetes distress was assessed using a 2-item screening instrument (DDS2) asking respondents to rate on a 5-point scale (1 = no distress and 5 = serious distress) the degree to which the following caused distress: (1) feeling overwhelmed by the demands of living with diabetes and (2) feeling that I am failing with my diabetes regimen. Scores were the average of the 2 items.^31^

^e^ Depression symptoms were assessed using a 2-item screening instrument (PHQ-2) asking respondents to rate on a 4-point scale (0 = not at all and 3 = nearly every day) the degree to which they had the following symptoms: (1) little interest or pleasure in doing things and (2) feeling down, depressed, or hopeless. Scores were the sum of the 2 items.^32^

**Table 2.** Baseline Characteristics for Phase 2 Participants^a^

Variable	No. (%)			
	Usual care (n = 47)	Mentoring from former mentee (n = 68)	Former mentee randomized to phase 2	
			Nonmentor (n = 69)	Mentor (n = 70)
Age, mean (SD), y	62.3 (6.8)	62.3 (6.9)	60.7 (7.6)	60.4 (6.4)
Male	100	97	100	100
Race/ethnicity, self-reported				
White	9 (19.2)	20 (29.4)	19 (27.5)	15 (21.4)
Black	37 (78.7)	45 (66.2)	45 (65.2)	52 (74.3)
Other^b^	1 (2.1)	3 (4.4)	5 (7.3)	3 (4.3)
Hispanic	4 (8.5)	1 (1.5)	4 (5.8)	3 (4.3)
Education				
<High school	2 (4.3)	2 (2.9)	4 (5.8)	6 (8.6)
High school	17 (36.2)	23 (33.8)	23 (33.3)	24 (34.3)
Some college	21 (44.7)	29 (42.7)	32 (46.4)	32 (45.7)
≥College	7 (14.9)	14 (20.6)	10 (14.5)	8 (11.4)
Partnered	22 (46.8)	36 (52.2)	35 (50.7)	36 (51.4)
Lives alone	9 (19.2)	18 (26.5)	28 (40.6)	20 (28.6)
Income, tertile				
Low (<$15 000/y)	11 (23.4)	16 (23.5)	13 (18.8)	20 (28.6)
Mid ($15 000 to <$40 000/y)	14 (29.8)	20 (29.4)	19 (27.5)	22 (31.4)
High (>$40 000/y)	14 (29.8)	14 (20.6)	31 (44.9)	15 (21.4)
Unreported (do not know or refused)	8 (17.0)	18 (26.5)	6 (8.7)	13 (18.6)
BMI, mean (SD)	31.0 (6.2)	32.3 (6.5)	34.0 (7.5)	32.1 (5.4)
General health history, mean (SD)				
Self-rated health^c^	3.3 (1.0)	3.5 (0.9)	3.3 (0.9)	3.3 (0.9)
Duration of diabetes, mean (SD), y	14.1 (9.2)	15.2 (10.9)	15.0 (11.7)	13.5 (7.6)
Antihyperglycemic medications				
Oral medications only	5 (10.6)	9 (13.2)	24 (34.8)	14 (20.0)
Insulin only	17 (36.2)	19 (27.9)	16 (23.2)	15 (21.4)
Insulin + oral medications	25 (53.2)	40 (58.8)	29 (42.0)	41 (58.6)
Clinical measures, mean (SD)				
HbA_1c_	9.7 (2.0)	9.2 (1.7)	8.9 (1.4)	9.4 (1.7)
LDL	84.3 (33.8)	93.3 (35.4)	90.9 (31.7)	88.6 (29.5)
SBP	134.8 (15.9)	140.5 (18.0)	137.1 (19.9)	138.8 (15.6)
DBP	77.8 (11.0)	78.5 (12.5)	79.6 (16.0)	79.7 (10.8)
DDS2, mean (SD)^d^	2.7 (1.3)	2.7 (1.1)	2.4 (1.1)	2.6 (1.1)
PHQ-2, mean (SD)^e^	1.8 (1.8)	1.9 (1.9)	1.7 (1.8)	1.5 (1.5)

Abbreviations: BMI, body mass index (calculated as weight in kilograms divided by height in meters squared); DBP, diastolic blood pressure; DDS, Diabetes Distress Scale; HbA_1c_, hemoglobin A_1C_; LDL, low-density lipoprotein; PHQ, Patient Health Questionnaire; SBP, systolic blood pressure.

SI conversion factors: To convert LDL to mmol/L, multiply by 0.0259; HbA_1c_ to proportion of total hemoglobin, multiply by 0.01.

^a^ The number in each group represents the number of participants analyzed.

^b^ Other is a convenience category that includes multiple races (checked more than 1 box), Asian, Native Hawaiian or Pacific Islander, Alaskan Native or American Indian, other (could be written in if desired but coded as other), and missing.

^c^ Self-rated health was assessed through the Short Form Health Survey, question 1 (SF-1): “In general, would you say your health is Excellent, Very Good, Good, Fair, Poor.” Scores range from 1 indicating excellent to 5 indicating poor.

^d^ Diabetes distress was assessed using a 2-item screening instrument (DDS2) asking respondents to rate on a 5-point scale (1 = no distress and 5 = serious distress) the degree to which the following caused distress: (1) feeling overwhelmed by the demands of living with diabetes and (2) feeling that I am failing with my diabetes regimen. Scores were the average of the 2 items.^31^

^e^ Depression symptoms were assessed using a 2-item screening instrument (PHQ-2) asking respondents to rate on a 4-point scale (0 = not at all and 3 = nearly every day) the degree to which they had the following symptoms: (1) little interest or pleasure in doing things and (2) feeling down, depressed, or hopeless. Scores were the sum of the 2 items.^32^

**Figure.**
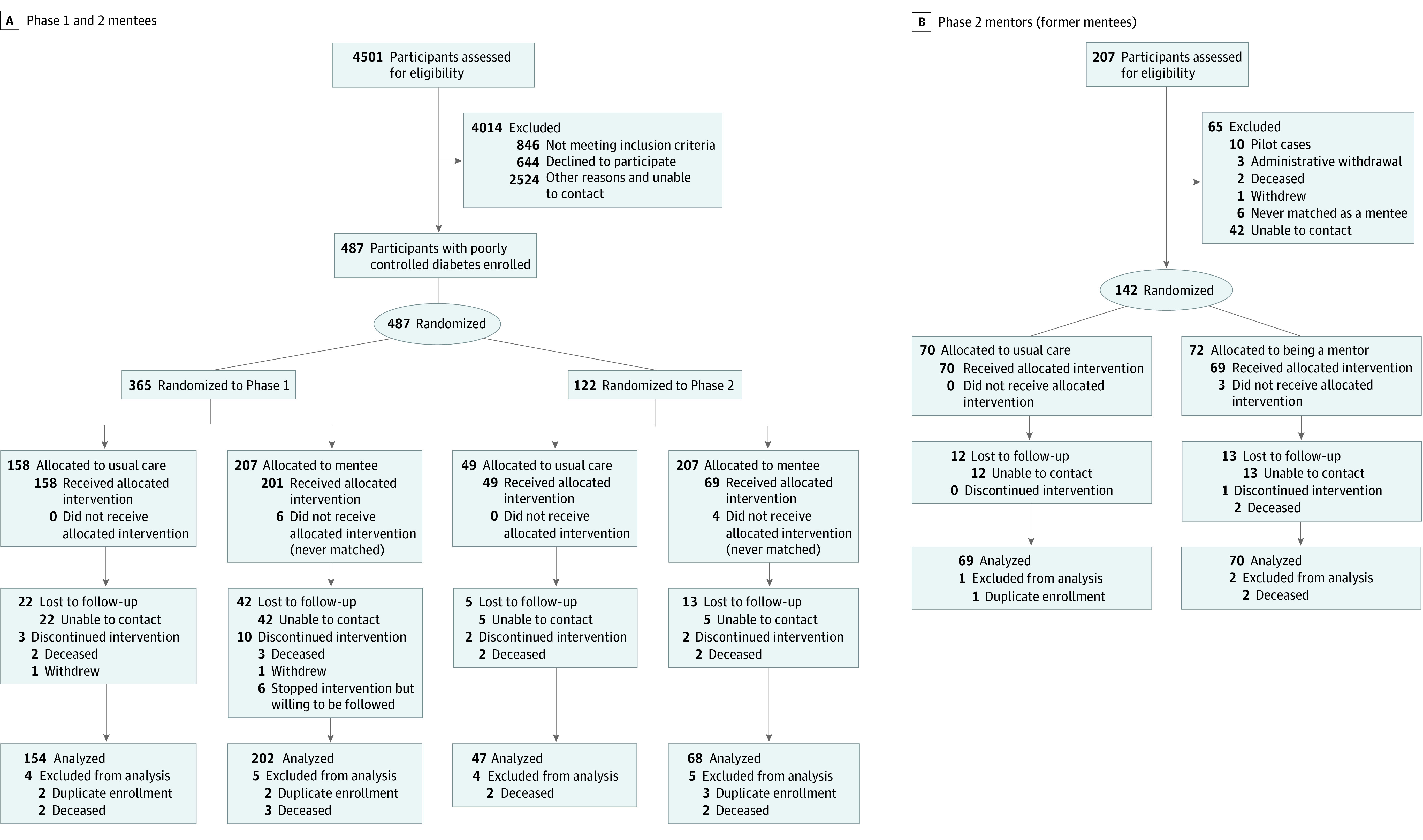
Participant Flow Diagram Multiple imputation was used to include people in the final analysis even if they were lost to follow-up. Only those who were unintentionally enrolled more than once and those who died were excluded.

In phase 1, compared with the intervention group, the usual care group had a greater mean baseline HbA_1c_ (9.8% vs 9.3%). The mean baseline HbA_1c_ was not different by arm in phase 2 or among phase 1 participants randomized to become a mentor or nonmentor. Demographic data and baseline LDL and BP levels were similar between treatment and control groups for all 3 analysis cohorts.

### Phase 1 Mentees vs Usual Care

At 6 months, the mean change in HbA_1c_ was −0.20% (95% CI, −0.46% to 0.06%) in the usual care arm and −0.52% (95% CI, −0.76% to −0.29%) for the intervention arm (*P* = .06) (Table 3). There was no difference in HbA_1c_ between arms at 12 months. The intervention did not affect BP, LDL, diabetes distress, or depressive symptoms. On enrollment, 58 people had an HbA_1c_ measurement less than or equal to 8%. Of these, 15 were in the usual care arm and 43 were in the intervention arm. In nonprespecified analyses, when we limited the analysis to those with a baseline HbA_1c_ greater than 8%, the mean change in HbA_1c_ was −0.32% (95% CI, −0.60% to −0.05%) for usual care and −0.75% (95% CI, −1.01% to −0.48%) for the intervention arm (*P* = .03). For the mentee arm compared with usual care, the odds of decreasing HbA_1c_ by 1% was 1.70 (95% CI, 1.01-2.86; *P* = .05).

**Table 3.** Changes in Phase 1 Outcomes From Baseline to 6 and 12 Months

Outcome	Usual care (n = 154)	Mentee (n = 202)	*P* value
**Primary outcome**^**a**^			
HbA_1c_ (95% CI)			
Baseline to 6 mo	−0.20 (−0.46 to 0.06)	−0.52 (−0.76 to −0.29)	.06
Baseline to 12 mo	−0.26 (−0.53 to −0.01)	−0.28 (−0.53 to −0.03)	.92
HbA_1c_ baseline >8% (95% CI)^b^			
Baseline to 6 mo	−0.32 (−0.60 to −0.05)	−0.75 (−1.01 to −0.48)	.03
Baseline to 12 mo	−0.39 (−0.67 to −0.12)	−0.48 (−0.76 to −0.19)	.68
**Secondary outcome**^**a**^			
LDL (95% CI)			
Baseline to 6 mo	−2.08 (−6.58 to 2.42)	−4.31 (−8.31 to −0.31)	.46
Baseline to 12 mo	−7.17 (−11.77 to −2.57)	−7.86 (−12.40 to −3.31)	.83
SBP (95% CI)			
Baseline to 6 mo	−3.45 (−6.23 to −0.66)	−0.87 (−3.48 to 1.74)	.18
Baseline to 12 mo	−3.75 (−6.60 to −0.91)	−2.33 (−4.92 to 0.27)	.47
DBP (95% CI)			
Baseline to 6 mo	−1.72 (−3.39 to −0.06)	−0.82 (−2.37 to 0.72)	.43
Baseline to 12 mo	−2.56 (−4.26 to −0.85)	−2.41 (−3.97 to −0.85)	.90
DDS2 (95% CI)			
Baseline to 6 mo	0.02 (−0.14 to 0.18)	−0.04 (−0.20 to 0.13)	.65
Baseline to 12 mo	−0.12 (−0.28 to 0.04)	−0.18 (−0.34 to −0.02)	.62
PHQ-2 (95% CI)			
Baseline to 6 mo	−0.05 (−0.28 to 0.18)	−0.04 (−0.26 to 0.18)	.94
Baseline to 12 mo	−0.12 (−0.35 to 0.11)	−0.17 (−0.39 to 0.04)	.72

Abbreviations: DBP, diastolic blood pressure; DDS, Diabetes Distress Scale; HbA_1c_, hemoglobin A_1C_; LDL, low-density lipoprotein; PHQ, Patient Health Questionnaire; SBP, systolic blood pressure.

SI conversion factors: To convert LDL to mmol/L, multiply by 0.0259; HbA_1c_ to proportion of total hemoglobin, multiply by 0.01.

^a^ Adjusted for baseline value and patient random effects.

^b^ Based on a subset analysis including patients with baseline HbA_1c_ greater than 8% (baseline was assessed after enrollment, and some patients who were recruited as poorly controlled based on prior medical records showed good control at baseline). This analysis included 139 and 159 patients in the usual care and mentee group, respectively.

### Phase 2 Mentoring From a Former Mentee vs Usual Care

The mean change in HbA_1c_ was −0.46% (95% CI, −1.02% to 0.10%) in the usual care arm and 0.08% (95% CI, −0.42% to 0.57%) for the mentoring from a former mentee arm (*P* = .16) (Table 4). At 6 months, compared with usual care participants, mentees who received mentoring from a former mentee showed statistically significant improvement in the Diabetes Distress Scale score of 0.10 points; 95% CI, −0.20 to 0.41 for the usual care arm vs −0.41 points; 95% CI, −0.68 to −0.14 for the intervention arm; *P* = .02). Similar to phase 1, effects did not persist. No other outcomes showed statistically significant differences.

**Table 4.** Changes in Phase 2 Outcomes From Baseline to 6 and 12 Months

Outcome	Usual care (n = 47)	Mentoring from a former mentee (n = 68)	*P* value
**Primary outcome**^**a**^			
HbA_1c_ (95% CI)			
Baseline to 6 mo	−0.46 (−1.02 to 0.10)	0.08 (−0.42 to 0.57)	.16
Baseline to 12 mo	−0.27 (−0.89 to 0.36)	−0.16 (−0.65 to 0.33)	.80
HbA_1c_ baseline >8% (95% CI)^b^			
Baseline to 6 mo	−0.67 (−1.30 to −0.04)	0.32 (−0.91 to 0.28)	.42
Baseline to 12 mo	−0.61 (−1.32 to 0.09)	−0.47 (−1.07 to 0.13)	.76
**Secondary outcome**^**a**^			
LDL (95% CI)			
Baseline to 6 mo	7.91 (−0.70 to 16.52)	−1.62 (−9.25 to 6.00)	.11
Baseline to 12 mo	5.04 (−4.10 to 14.18)	−4.91 (−12.81 to 2.99)	.11
SBP (95% CI)			
Baseline to 6 mo	1.47 (−3.53 to 6.47)	0.19 (−4.05 to 4.42)	.70
Baseline to 12 mo	−1.69 (−6.88 to 3.51)	1.39 (−2.77 to 5.55)	.37
DBP (95% CI)			
Baseline to 6 mo	0.23 (−2.81 to 3.26)	−1.37 (−3.93 to 1.18)	.43
Baseline to 12 mo	−1.09 (−4.20 to 2.03)	−1.76 (−4.28 to 0.76)	.74
DDS2 (95% CI)			
Baseline to 6 mo	0.10 (−0.20 to 0.41)	−0.41 (−0.68 to −0.14)	.02
Baseline to 12 mo	−0.13 (−0.45 to 0.19)	−0.02 (−0.31 to 0.26)	.62
PHQ-2 (95% CI)			
Baseline to 6 mo	−0.32 (−0.77 to 0.14)	−0.26 (−0.67 to 0.15)	.86
Baseline to 12 mo	−0.46 (−0.94 to 0.02)	−0.09 (−0.52 to 0.34)	.25

Abbreviations: DBP, diastolic blood pressure; DDS, Diabetes Distress Scale; HbA_1c_, hemoglobin A_1C_; LDL, low-density lipoprotein; PHQ, Patient Health Questionnaire; SBP, systolic blood pressure.

SI conversion factors: To convert LDL to mmol/L, multiply by 0.0259; HbA_1c_ to proportion of total hemoglobin, multiply by 0.01.

^a^ Adjusted for baseline value and patient random effects.

^b^ Based on a subset analysis including patients with baseline HbA_1c_ greater than 8% (baseline was assessed after enrollment, and some patients who were recruited as poorly controlled based on prior medical records showed good control at baseline). This analysis included 39 and 50 patients in the usual care and former mentee group, respectively.

In nonprespecified analyses, when we compared the change in HbA_1c_ between those whose mentor had successfully decreased their HbA_1c_ by greater than or equal to 1% in phase 1 to those who had not, those mentored by a past successful mentee dropped their HbA_1c_ by 0.28% (95% CI, −0.89% to 0.34%) compared with an increase in HbA_1c_ of 0.76% (95% CI, −0.05% to 1.57%) in those who received mentoring from a past unsuccessful mentee (*P* = .05).

### Phase 2 Mentors vs Nonmentors

Becoming a mentor in phase 2 did not prove beneficial to former mentees. Six months after being randomized to become a mentor or not, both those randomized to being a mentor and nonmentors had increases in HbA_1c_ (0.1% and 0.3%, respectively, *P* = .54).

### Adverse Events

No SAEs or AEs were deemed to be related to the study. No participant was removed from the study as a result of an SAE or AE. The Data Safety Monitoring Board did not request that any additional analyses be performed to evaluate hypoglycemic events.

## Discussion

This randomized clinical trial found that, compared with usual care, veterans with diabetes and poor glycemic control receiving mentoring from a veteran whose diabetes was once in poor control but now in good control marginally improved HbA_1c_ after the 6-month intervention. This finding was not statistically significant at *P* < .05. Gains were not sustained at 1 year. In addition, receiving mentoring from a former mentee did not lead to improvements in HbA_1c_ and may have even led to worsening of glycemic control if the “mentor” had not improved their own control when a mentee themselves. Compared with former mentees who did not themselves become mentors, serving as a mentor also did not lead to improvements in HbA_1c_. The intervention had no further benefits on BP, lipids, diabetes distress, or depression symptoms. To our knowledge, this is the first study to evaluate benefits of peer mentor models beyond 6 months and to assess a potentially more sustainable model in which previous mentees serve as mentors.

Our study differed from other peer mentor studies because of the lower-touch hour-long mentor training curriculum. Mentors in our study received a 1-time, 1-hour training session with monthly reinforcement sessions from research staff. Heisler et al^24^ provided a 3-hour training session plus optional group sessions, and Thom et al^22^ had mentors attend 36 hours of training and take an exam. While the intervention was purposefully designed this way to make it easier to implement, a higher-touch intervention may have led to improved diabetes-related outcomes.

The marginal effects seen at 6 months did not persist at 1 year regardless of starting HbA_1c_. This outcome is comparable with other programs that address behavior change, such as diabetes self-management education, which show a diminishing effect after the intervention ends.^36^ One possible explanation for the lack of sustained effects on glycemic control is the loss of support for peer pairs to stay in touch. Our peer mentor model, like others, supported mentors with financial incentives and monthly phone calls from research staff.^22,23,24,31^ The financial incentive of $20 a month to call the mentee weekly plus the monthly training enforcement and contact from the study staff did not continue after 6 months. Future peer mentor interventions may benefit from supporting longer-term interactions.

Heisler et al^24^ found that a reciprocal peer support intervention improved HbA_1c_. Given these results, we anticipated seeing benefits on glycemic control in participants who became mentors. Not only did we not observe benefits, having a mentor who did not improve their own control in phase 1 was associated with worsening control for their mentees. Use of the term *mentor* may have been detrimental because, anecdotally, some phase 2 mentees noted that their own control was better than that of their mentors. Transitioning former mentees to mentors is 1 way of maintaining sustainability of peer support models; however, using former mentees who improved glycemic control may be the best approach to obtain optimal outcomes.

We did not find any further effects of our peer mentor intervention on BP, lipids, diabetes distress, or depression symptoms. Our results reinforce the findings from other studies, which also found no significant changes in these outcomes.^22,24^ Of note, the study was not powered to detect differences in BP or lipids, and participants’ baseline values were fairly well controlled. In addition, mentors were not specifically skilled or trained to address mentees’ diabetes distress or depression symptoms. Our qualitative analysis of mentor-mentee pairs indicated that multiple comorbidities, especially poor mental health, hindered the mentor-mentee relationship.^29^ A peer mentor model may not be appropriate for patients also dealing with severe mental health issues.

### Limitations

This study has limitations. First, we conducted the study at 1 VA medical center in a mostly Black male population. The findings may not be generalizable outside of the VA community. A peer mentor study conducted in public health clinics with a more varied patient population did show improvements in glycemic control.^22^ Second, we did not succeed at only enrolling veterans with very poor control; however, effects did not persist even for those with an HbA_1c_ level greater than 8% at baseline. Third, the training of mentors by design was short and limited, and we did little to select naturally inclined mentors. Our qualitative results indicate that providing additional mentor training to build structure into mentor-mentee interactions, choosing mentors who are inherently good at providing coaching, and not targeting mentees struggling with challenging comorbidities could potentially enhance the impact of the program.^29^

## Conclusions

In this randomized clinical trial, there was no difference between a peer support intervention vs usual care for improving glycemic control in patients with diabetes. Initial gains were marginal and not maintained. However, several findings from ad hoc analyses indicated that initial gains were more pronounced in patients with starting HbA_1c_ levels above 8% and that mentoring from a former mentee who did not improve while a mentee could lead to worse outcomes. Future studies to determine how best to facilitate mentor and mentee engagement and optimal practices to create long-term sustainable peer-mentoring models may be warranted.

## Notes

Supplement 1. **Trial Protocol**

Supplement 2. **Data Sharing Statement**
