# Supplementary material for: Effect of Peer Mentors in Diabetes Self-management vs Usual Care on Outcomes in US Veterans With Type 2 Diabetes: A Randomized Clinical Trial
Source: JAMA Netw Open. 2020 Sep 11;3(9):e2016369. doi: 10.1001/jamanetworkopen.2020.16369 (PMC7489832; doi:10.1001/jamanetworkopen.2020.16369)
Supplement: Supplement 1. — Trial Protocol [file jamanetwopen-e2016369-s001.pdf]

Human Studies Subcommittee (IRB II)
Department of Veterans Affairs Medical Center
Research and Development Service
3900 Woodland Avenue • Philadelphia, PA 19104 • 215-823-3003 • Fax: 215-823-5171

IRB APPROVAL - Amendment

Date: January 13, 2016
From: Terri M. Laufer, MD, Vice Chairperson
Investigator: Judith A. Long, M.D.
Protocol: Using Peer Mentors to Support PACT Team Efforts to Improve Diabetes Control (CEPACT)
ID: 01373 Prom#: 0013 Protocol#: N/A

The following items were reviewed and approved through Expedited Review:

- Amendment - Expand enrollment by 115 - 69 poorly controlled diabetic veterans and 46 mentors (12/11/2015)

Expedited Approval [Expedited under Federal Regulation: 45 CFR 46.110(b)(2) / VA Regulation: 38 CFR 16.110(b)(2)] was granted on 01/13/2016. This Expedited review will be reported to the fully convened Human Studies Subcommittee (IRB II) on 01/20/2016.

The following other committee reviews are scheduled:
Research & Development Committee [02/02/2016]

The Corporal Michael J. Crescenz VAMC IRB is not connected with, has no authority over, and is not responsible for human research conducted at any other institution, except where a Memorandum of Understanding specifies otherwise. Separate consent forms, initial reviews, continuing reviews, amendments, and reporting of serious adverse events are required if the same study is conducted at multiple institutions.

CMCVAMC Specific Protocol Summary Content Requirements for IRB Committee Review	CMCVAMC IRB CMC VA Medical Center Institutional Review Board

A. **Protocol Title**
   1. **Full Protocol Title:** Using Peer Mentors to Support PACT Team Efforts to Improve Diabetes Control (CEPACT)

   2. **Date of Protocol Summary and Version #: Date 12/15/2015; Version # 7**

B. **Principal Investigator's Full Name and Degree:** Judith A. Long, MD

APPROVED by CMCVAMC IRB 2 Date: 1/13/16

C. **Co-Investigator's Full Name and Degree**:

D. **Financial Sponsor** (Provide the name of the agency, organization, company or person providing funds for the research study.) **HSR&D**

E. **Grant (Provide the** name of individual who holds the grant and the grant number, if applicable.) **N/A**

F. **Protocol Number** (Provide the financial sponsor's protocol number, if applicable.) **N/A**

G. **Institution(s) responsible for the project:**
   1. For single-site studies - CMCVAMC is the only institution involved. Yes X No ☐
   2. For multi-center studies.
      2.1. CMCVAMC is the Coordinating Center in which the PI is the lead investigator. Yes ☐ No ☐ N/A ☐
      2.2. Provide the name of the Coordinating Center. Yes ☐ No ☐ N/A ☐
      2.3. List the name of the other sites involved.
      2.4. Provide the FWA numbers for each of the other sites involved.

THE FOLLOWING INFORMATION MUST BE CMCVAMC-SPECIFIC, THAT IS, SPECIFIC TO WHAT WILL BE DONE WITH CMCVAMC-RECRUITED VETERANS.

H. **Background and Significance:** (Describe succinctly and clearly the past findings which justify the plan for this project. A summary of the relevant literature in the area of interest and reports of previous studies should be included.)

Peer support and particularly mentors have been shown to help African American diabetic veterans with poor diabetes control improve their glucose control. Ongoing studies are testing whether mentors can help non-veterans and populations including a larger sample size of women. Peer mentors may be particularly effective in a VA setting where many patients lack social support and a culture of camaraderie is strong. However, it is unknown if improvements persist once mentoring stops, how to best sustain such programs and whether peer support models would be effective in veterans from all ethnic racial groups.

Purpose of the Project: (Clearly provide the purpose of this research project.)

In this study, we propose a randomized controlled trial of poorly controlled diabetic veterans in which poorly controlled veterans of all race/ethnicities first enroll as mentees in a program for diabetic veterans and then as mentors to test a model of sustainability. In addition, given a growing literature that being a mentor is good for your health besides from creating sustainability for the program it may help sustain

effects. This study will have 4 arms: 1. Usual care (enrolled at two different time points); 2. Peer mentoring where mentors are veterans who were once in poor control but are now in good control; 3. Peer mentoring where the mentoring comes from former mentees from arm 2 (mentoring FFM); and 4. Former mentees from arm 2 not randomized to becoming a mentor. Ultimately we aim to create programs that can serve to sustainably support VA Patient Aligned Care Team (PACT) efforts.

Describe the Research Questions or Hypotheses (that is, what questions are you trying to address by conducting the research.)

Our primary aims are as follows.

1. Test the effectiveness of a peer mentor model in a mixed race population of poorly controlled diabetic veterans.
   H1: Compared to usual care, veterans in the peer mentor arms will have improved hemoglobin (HbA_{1c}), blood pressure, LDL levels, DM quality of life, and depression scores regardless of race or ethnicity at the end of the intervention (tested at 6 months) and effects will persist (tested at 12 and 18 months).
2. Test the effectiveness of using former peer mentees as peer mentors.
   H2: Compared to usual care, veteran who receive peer mentoring from former mentees will have improved hemoglobin (HbA_{1c}), blood pressure, LDL levels, DM quality of life, and depression scores at the end of the intervention (tested at 6 months) and effects will persist (tested at 12 and 18 months).
3. Assess the effects of becoming a mentor on those who were originally mentees.
   H3: Compared to past mentees who are not randomized to becoming a mentor, past mentees randomized to becoming a mentor will have better glucose control, blood pressure, LDL levels, DM quality of life, and depression scores.
4. Conduct a rigorous qualitative evaluation examining in-depth the mentor-mentee relationship, the transition to becoming a mentor, qualities of a successful mentor, and factors relevant to broader program implementation.

Secondary Aims:

5. In those randomized to being a mentee, explore mentor characteristics associated with improved HbA_{1c}. Predictors to be evaluated include past mentoring dose of the current mentor, the mentor's past change in HbA_{1c}, the mentor's starting HbA_{1c}, current mentoring dose provided by the mentor, mentee's evaluation of the mentor, and mentor's depression score at baseline.

I. **Primary Outcome Variable(s):** (Define the primary outcome variable(s) used to support the study objectives (e.g. if the objective is to show that treatment A is superior to treatment B in the treatment of subjects with essential hypertension, the primary outcome variable is blood pressure measurement.)

   The primary outcome variable will be change in glucose control as measured by the HbA1c assessed at baseline, 6 months,12 months and 18 months.

J. **Secondary Outcome Variable(s):** (Define the secondary outcome variables. Such measured variables should also include the timing of measurement.)

   Secondary outcome variables will include blood pressure, serum LDL, diabetes quality of life measured by the self reported Diabetes Distress Scale, and depression assessed with the PRIME-MD Patient Health Questionnaire (PHQ-9). All secondary outcomes will also be measured at baseline, 6 months and 12 months.

K. **Study Design and Methods:**

1. Is this a clinical trial? X YES ☐NO
   1.1. If yes, what type? Check all that apply.
   ☐ **Phase I** ☐ **Phase II** **X Phase III** ☐ **Phase IV**
   1.2. If yes, this study must be registered on Clinicaltrials.gov.

2. **Design**
   2.1. What research methods will be used in the project? Check all that apply.

XSurveys/Questionnaires	X Interviews	☒ Audio Taping
☐ Behavioral Observations	☐ Chart Reviews	☐ Video Taping
☐ Focus Groups	X Randomization	☐ Double-Blind
XControl Group	☐ Placebo	☐ Withhold/Delay Treatment
XSpecimen Collection	☐ Deception	X Telephone Survey
☐ Other (Describe)		

   2.2. Describe how randomization or other treatment assignment will be made.

After informed consent is obtained and the baseline interview is complete, participants will be randomized. Randomization will be a staged process (see figure 1). For both steps, randomization will be conducted by the study statistician using a random number generator and via permuted block randomization with variable block size to force balance among treatment group assignments after every block of participants is enrolled.

At stage 1, interested participants will be randomized to usual care or peer mentoring. At stage 2, interested participants will be randomized to usual care or peer mentoring from a former mentee (FFM). In addition, former mentees with be randomized to either becoming a mentor or usual care (i.e. mentee assigned or no mentee assigned).

To date we have been randomizing patients in a 1:1 manner. After receiving IRB approval for this modification we will change to 2:1 randomization (2 to an intervention arm for every 1 enrolled into usual care). See below for exact numbers.

Figure 1 Randomization

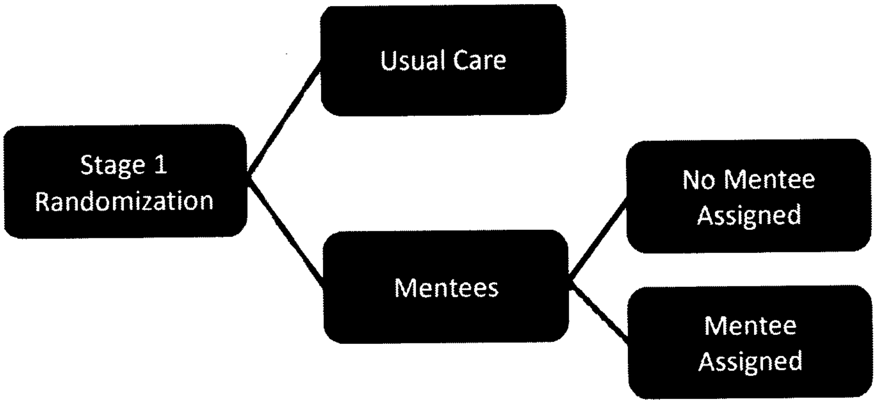

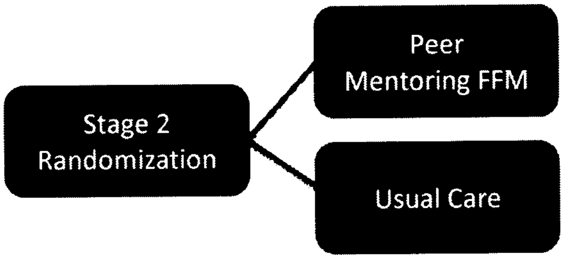

2.3. For retrospective research studies, provide the "look-back" period. (e.g., December 1, 1999 through December 31, 2008.)

3. **Study Duration**

3.1. Provide the estimated length of time to enroll all subjects and complete the study.

We want to enroll a total of 492 poorly controlled diabetic veterans and 206 mentors with currently good glucose control. In stage 1 there will be 206 poorly controlled diabetics in the peer mentoring arm and 206 mentors. Mentors who withdraw from the study will be replaced by additional mentors to ensure that all 206 poorly controlled diabetics in the peer-mentoring arm have mentors. In stage two there will be 144 mentors (former mentees) of which 72 will be randomized to being a mentor and 72 will be randomized to usual care. In addition, there will be 128 poor controlled diabetics 56 of whom will be in usual care and 72 of whom will be randomized to receiving peer mentoring from FFM. We project being able to enroll 7-8 new poorly controlled diabetics per week with two research assistants. Even considering the staggered start times, we anticipate it will take 14-16 months to complete enrollment and another 18-24 months to complete follow-up for a total of 38-40 months. After that we anticipate it will take an additional 1-2 months to complete the analysis. See sample size section for further description of required sample.

3.2. Explain the expected duration of subject participation including any follow-up.

The active time for each participant in the study will be roughly 18-24 months depending on which arm they are randomized to. 12-18 months of face-to face visits with an additional 6 months after the last in-person visit where each participant's electronic medical record (EMR) will be under electronic review. Figure 2 depicts the flow of participants through the study.

Figure 2: Flow of Participants Through Study

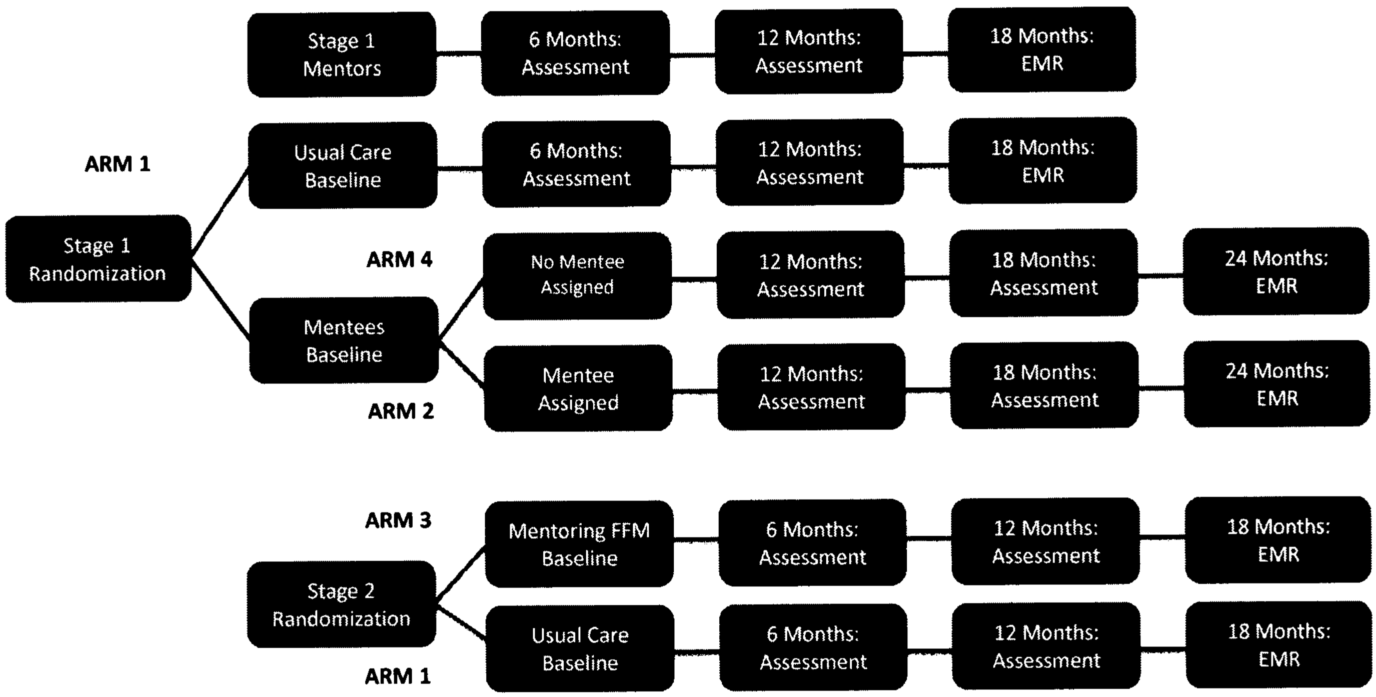

3.3. Specify the projected date of completion of the proposed study.

Enrollment started September 27, 2012 and we anticipate completion of the study by December 31, 2015

4. **Drug Information** (If not applicable state, "Not Applicable.") **N/A**

4.1. Specify if the drug or biological agent is:
- 4.1.1. FDA approved
- 4.1.2. Used for off-label purposes
- 4.1.3. Not yet FDA approved.

4.2. Include the FDA Investigational New Drug (IND) number for all non-FDA approved and off-label drugs, biological agents or nutritional supplements. If not applicable state, "Not Applicable."

4.3. Provide all relevant information about the drug

4.4. Explain any wash-out periods, rescue medications permitted and any type of medications not permitted while enrolled in the study.

4.5. Describe blinding and un-blinding procedures.

4.6. Include the dosage, route of administration, previous use, and the safety and efficacy information on any drug used for research purposes.

4.7. Describe rationale for the dosage in this study.

4.8. Justify why the risks are reasonable in relation to anticipated benefits and/or knowledge.

4.9. Describe where drug preparation will be done.

4.10. All drugs for CMCVAMC subjects must be dispensed through the VA investigational pharmacy.

4.11. Describe where the study treatment will be administered.

4.12. Describe plan for tracking a non-compliant treatment study subject.
4.13. Summarize any pre-clinical data.
4.14. Describe the process for the storage, security, dispensing and return of an investigational drug.

5. **Investigational Device** (If not applicable state, "Not Applicable.") **N/A**
   5.1. The Investigational Device Exemption (IDE) number must be submitted for all significant risk devices and if an IDE exists for a non-significant device.
   5.2. Significant Risk or Non-significant Risk - If a device is not approved by the FDA, specify whether or not the sponsor has determined this device to be a "significant risk" or "non-significant risk" as defined by the FDA.
   5.3. Provide all relevant information about the device.
   5.4. Describe blinding and un-blinding procedures.
   5.5. Specify if device is:
      5.5.1. FDA approved
      5.5.2. Used for off-label purposes
      5.5.3. Not yet FDA approved.
   5.6. Explain if the investigational device will be delivered and/or stored by the Principal Investigator or Pharmacy Services.
   5.7. Describe the process for the storage, security, dispensing and return of an investigational device.
   5.8. For research involving an investigational device, describe the SOP or plan for device control.
   5.9. Address how the device will be stored in such a way that only research staff associated with the protocol will have access to the device.
   5.10. Describe measures that will be put into place to ensure that the device will only be used in participants of this research protocol.

L. **Does this project involve international research?** ☐YES **XNO**
   1. *For further instructions refer to VHA Directive 2005-050, Requirements for Conducting VA-Approved International Research Involving Human Subjects, Human Biological Specimens, or Human Data*
   2. *VHA Handbook 1200.05 definition of international research - VA international research is any VA-approved research conducted at international sites (not within the United States (U.S.), its territories, or Commonwealths); any VA-approved research using either human biological specimens (identified, de-identified, or coded) or human data (identified, de-identified, or coded) originating from international sites; or any VA-approved research sending such specimens or data out of the U.S. (see par. 56).* ***NOTE:*** *For the purposes of this Handbook, research conducted at U.S. military bases, ships, or embassies is not considered international research.*

M. **Study Procedure**
   1. **Study Procedures**
      1.1. Outline all study procedures - (*If necessary, include a table or flow chart, showing the schedule of the procedures and interactions. Distinguish between interventions that are experimental and carried out for research purposes vs. those that are considered standard of care. Routine procedures that are performed solely for research purposes should also be identified.)*

Overview: We will perform a randomized controlled trial with four arms. The aim is to create a program that can augment PACT efforts.

Patients and Settings: We will enroll veterans with diabetes mellitus (DM) who receive their care from the CMC VA Medical Center (CMCVAMC). Veterans between the ages of 30 and 75 years of age -- patients most likely to reap the benefits of gaining control -- will be eligible for participation. There will be 4 arms: 1. Usual care (enrolled at two different time points); 2. Peer mentoring where mentors are veterans who were once in poor control but are now in good control; 3. Peer mentoring where the mentoring comes from former mentees from arm 2 (mentoring FFM); and 4. Former mentees from arm 2 not randomized to becoming a mentor. To ensure that we are targeting those most likely to benefit from improving control we enroll diabetics with persistently poor DM control. Persistently poor DM control will be defined as having an HbA1c > 8% on 2 different occasions in the course of 24 months, with at least 1 measure within 3 months of enrollment. Mentors for arm 2 will have previously been in poor control and are now in good control, defined as having an HbA1c of > 8% in the past 3 years and an HbA1c ≤ 7.5% within 3 months of enrollment. They will also, when feasible, be the same race, sex, and age (± 10 years) as their mentee. When possible, if the mentee is on insulin we will seek out a mentor on insulin. These criteria for selecting both mentees and mentors have led to successful mentoring partnerships in our prior work. Patients will be identified by the Center for Evaluation of PACT (CEPACT) through VISN 4 Data Warehouse (VDW). With IRB approval, CEPACT will extract administrative, clinical, and laboratory data from the VDW on an ongoing basis. The VDW is updated regularly and thus an excellent source of up-to-date patient information. Identified patients will be sent a letter describing the study and then called. The PI has used this method to identify veterans with DM on many occasions and routinely is able enroll between 60-70% of those contacted. Potential mentors for arm 2 will be identified and approached in a similar manner. Veterans who agree to participate will be invited into the CMC VAMC to complete a consent procedure, baseline survey, and have their blood drawn to determine their HbA1c at enrollment. All enrollees and mentors will receive, regardless of the arm, $50.00 for each visit requiring a blood draw and survey completion (baseline, 6 months, 12 months, and depending on arm, potentially 18 months).

At the end of 6 months, those randomized to receiving a mentor in stage 1 will be re-randomized to either becoming a mentor (arm 2) or not becoming a mentor (arm 4). Those who do not become a mentor will continue to be followed. Those randomized to being a mentor will become mentors for arm 3 participants. For arm 3, matching will be tailored to the best of our ability since the pool of mentors will be limited to those who were previously mentees. We have built in over-enrollment into stage 1 to allow for some loss to follow-up and selective removal of participants who might be inappropriate mentors. Potential mentees for stage 2 will be identified and contacted in the same manner as those identified for stage 1. Those interested in participating will be randomized to usual care or mentoring from FFM. As with stage 1, participants will receive $50 to reimburse them for their time for all required in person visits.

Procedures: As mentioned there will be 4 arms. Randomization into each arm is described above.

Arm 1 Usual Care: They will complete all planned surveys, interviews and blood draws (baseline, 6 months, 12 months). In addition, all participants' medical health record will be under electronic review for an additional 6 months after their last visit. They will be notified of their starting HbA1c and the American Diabetes Association (ADA) and VA recommended goals for HbA1c. No additional interventions will be provided. All participants will be called at one month and three months to check in and assess for hypoglycemic events (based on our past previous work we anticipate that these events will be rare and not differ by arm).

Arm 2 Peer Mentoring: In addition to being notified of their starting HbA1c and ADA and VA recommendations for HbA1c, arm 2 will be told that within the next 1-3 weeks we will find them a peer mentor who will start calling them. They will be told that they are expected to talk with the mentor at least once a week for 6 months but they can talk as much as they want. We will also

explain that we will not be setting up a face-to-face introduction but that it is fine for the two of them to meet in person if they desire. Finally, participants will be reminded that in six months time they may become a mentor. We will train them to be mentors and provide them with a mentee who is in poor diabetes control.

After being randomized to the peer mentor arm we will identify potential mentors and contact people until an eligible mentor is found. Mentors will participate in an hour long one-on-one training informed by motivational interviewing techniques. All training materials have been developed and include instruction on learning the mentee's story, understanding the mentee's motivations, helping the mentee identify the differences between their behaviors and goals, and helping the mentee identify a realistic plan for goal achievement. Open ended questions are encouraged and modeled. Mentors are also taught how to follow-up and assess progress. Sample questions will be provided. However, mentors are also encouraged to draw on their own experiences. Once a month, peer mentors will be contacted to provide training reinforcement and asked about their interactions with their mentee. Peer mentors will be given the phone number of their mentee and informed that they will receive $20 per month for speaking at least once a week. Whether they spoke will be determined by self-report.

At the end of 6 months, at the first follow-up visit mentees will be randomly assigned to become a mentor or to not become a mentor. Those selected to become a mentor will be trained how to be mentors and told that we will contact them in the next 1-3 weeks with a mentee. They will then be asked to perform all of the activities that their mentor performed for them. Like their mentors, they will also receive $20 per month for talking at least once a week.

Arm 3 Peer Mentoring FFM: As with the other arms the participants will be notified of their starting HbA1c and ADA and VA recommendations for HbA1c. To the best of our ability, Arm 3 will be matched with a mentor race, sex, age (± 10 years), and insulin use. The mentor will come from Arm 2 and initiate phone contact.

Arm 4 Former Mentees not Randomized to Becoming a Mentor: Arm 2 participants not randomized to becoming a mentor will be followed in the same manner as arm 2 participants randomized to becoming a mentor.

Data Collection: All participants (mentors and mentees) will complete a baseline survey, a short call at 1 and 3 months, a 6 month survey, a 12 month survey, a blood draw at baseline, 6 months, and 12 months and will have their medical health record under electronic review for an additional 6 months after the last in-person visit. Those randomized to arm 2 and further randomized to arm 4 will receive additional short calls at 7 months and 9 months and have one additional in person visit with blood draw at 18 months. A sample of mentors and mentees from stage 1 and stage 2 will be asked to complete qualitative interviews baseline, 6, 12, 18 months. Table 1 identifies data to be collected at each time point and the source of the data.

Table 1: Measurement Schedule

	Baseline	1 M	3 M	6 M	12 M	18 M	24 M
**Dependent Variables**							
HbA$_{1c}$ (blood)	x			x	x	x	
Blood Pressure (in person measurement)	x			x	x	x	
Direct LDL (blood)	x			x	x	x	
DM quality of Life (self-report) - Distress	x			x	x	x	
Depression Symptoms (self-report) - Mood	x			x	x	x	
**Intervening Variables of Interest**							
Social Support	x			x	x	x	
Self-Efficacy (self-report)	x			x	x	x	
Perceived Benefits and Barriers (self-report)	x			x	x	x	
Self-management Behaviors (self-report)	x			x	x	x	
Past Mentoring Dose Received by Current Mentor (Past records)	x						
Mentor's past Change in HbA$_{1c}$ (Past study records)	x						
Mentee's assessment of mentor quality (self-report)				x	x	x	
**Potential Confounders**							
Demographics (self-report)	x						
Attachment Style (self-report)	x						
Perceived Need for Support (self-report)	x			x	x	x	
BMI (in person measurement)	x			x	x	x	
DM Health History (self-report)	x						
General Health History (self-report)	x			x	x	x	
Potential Contamination (self-report)				x	x	x	
**Hypoglycemic Symptoms** (self-report)	x	x	x	x	x	x	
**Qualitative Perceptions of Program** (self-report)	x			x	x	x	
**Medical Record Review** (Electronic medical record)**						x	x

* Current Mentoring Dose Provided by Mentor will be assessed monthly. For Ease of Presentation we did not include all collection times.

** Exact timing of EMR review depends on arm – only reviewed once either at 18 or 24 months.

For self-reported data we will use validated survey questions developed by the PI or other researchers in the field. The research coordinator will be trained to measure BP and pulse rate at each visit using an automatic digital blood pressure monitor on the non-dominant arm after the subject has been seated for 5 minutes. An average of two measurements will be used for analyses. We will evaluate both change in systolic and diastolic BP as well as the percent of patients in each arm with ADA target BP (BP < 130/80 mmHG). Height and weight will be measured using standard procedures with a beam balance and a strain-gauge digital scale, respectively, and used to calculate body mass index (BMI).

We will conduct the qualitative interviews with the aim of understanding how, in terms of the outcome HbA$_{1c}$, successful mentor-mentee pairs differed from those who were not successful. Interview guides will be informed by *adult learning theory.* We will use semi-structured interviews. Open-ended questions will be followed by probes to prompt detail and ensure that a broad range of possible responses are elicited. Using constant comparison techniques, we will modify scripts in real time as new and unexpected themes emerge.

1.2. Explain if and how the follow-up of subjects will occur.

Follow-up will occur at 1 and 3 months post enrollment with a brief phone call, and at 6 months and 12 months post enrollment at the CMC VA Medical Center. Additional short calls at 7 and 9 months and an additional in person 18 month visit will also occur for those in arm 2 and 4. After follow-up in person visits are complete, all enrolled participants' medical health record will be under electronic review for an additional 6 months.

1.3. Describe where, how and who will be conducting study procedures.

Consent, study surveys and interviews, blood pressure measurements, and weight at baseline, 6 months, 12 months and when appropriate 18 months will occur at the CMC VA Medical Center and be performed by a project manager or research assistant.

One and three month hypoglycemic assessments will be assessed over the phone by a project manager or research assistant.

Evaluations of the serum HbA1c and LDL will be performed by the CMC VA Medical Center lab.

Electronic review of medical health records will occur at the CMC VA Medical Center on the VA server network.

1.4. If a survey study, specify the estimated amount of time that subjects will need to complete the questionnaires/tools.

The below table estimates the amount of time each step will require for data collection. The amount of time spent on mentoring calls is not determined by the study.

Table 2: Data Collection Step, Time to Complete and Mode of Collection

Procedure	Estimated Time to Complete	Number of Times to Perform
Consent	30 minutes	1 (in person)
Baseline Survey	45 minutes	1 (in person)
Lab tests	10 minutes	3-4 (in person)
Hypoglycemic Symptoms	5 minutes	2 (phone call)
Follow-up Survey	30 minutes	2-3 (in person)
Qualitative Interviews	20 minutes	2-3 (in person)
Health record review	15 minutes	1 (electronically)
Mentor Training	60 minutes	1 (in person)
Reinforcement Calls	10-15 minutes	5 (phone call)
**Estimated Total Active Time for Participants**		
Arm 1	175 minutes (≈ 3.0 hours)	
Arm 2	385 minutes (≈ 6.5 hours)	
Arm 3	215 minutes (≈ 3.5 hours)	
Arm 4	385 minutes (≈ 6.5 hours)	
Mentors for Arm 2	325 minutes (≈ 5.5 hours)	

Based on experience, consent will take about 30 minutes and the baseline survey will take approximately 45 minutes. Follow-up visits will take about 30 minutes for survey completion and 20 minutes for the completion of qualitative interviews. The lab usually expedites tests for research and we expect patients will need to spend approximately 10 minutes getting their blood

drawn. The total time commitment for consent and data collection is estimated to be between 3 and 7 hours depending on the arm the person is in. However, given the time commitment we will reimburse participants $50 for each in person visit to cover time and transportation costs and an additional $40 for qualitative interviews if they agree to complete the qualitative interview portion of the study.

1.5. If a blood draw, specify the amount of blood to be drawn in milliliters and in teaspoonfuls or tablespoonfuls and specify how often and where the blood will be drawn.

Blood draws will occur at the CMC VAMC lab. We anticipate a maximum of 5ccs or 1 teaspoon of blood will be required for each in-person visit at baseline, 6 months, 12 months and when appropriate 18 months..

2. **Data Collection** (Include all questionnaires and survey tools with the submission.)

2.1. Provide

2.1.1. the mode of data collection, e.g. telephone, in-person, questionnaire, interviews,

Data will be collected mostly by in-person survey, interview and lab tests. In addition, mentors will receive short reinforcement calls each month at which time we will collect data about how many times the mentor talked to the mentee in the month. Hypoglycemic symptoms will be assessed by phone at 1 and 3 months (additionally at 7 and 9 months for arms 2 and 4) . Electronic medical records will be reviewed 6 months after the last in person visit. See Table 1 and 2 for details.

2.1.2. the precise plan for how data is to be collected or acquired

Surveys will be read to participants and answers will either be directly input into a computer database or written onto paper forms and then transferred to a data base at a later time. The data base will reside on VA servers behind the VA firewall and will never be removed from the server. Paper records will be stored at CHERP behind an electronically locked entrance, a key-locked door and in a key-locked cabinet.

Qualitative interviews will also be read to participants and answers will be voice recorded. Interview data will be stored on the VA server and will be behind VA firewalls This data will be temporarily shared with Alpha Transcription, and University of Pennsylvania's Mixed Methods Research Lab, transcribing and coding respectively. Both companies are approved vendors with appropriate VA approved data security in place.
Data about the number of calls per month made by the mentor will be collected over the phone, and again responses will either be entered directly into a database or first onto paper and then into the database.

Data about hypoglycemic symptoms will be collected over the phone and responses will either be entered directly into a database or first onto paper and then onto the database.

Lab results will be obtained from the lab or via CPRS and entered into the secure research database.

Charts will be abstracted for additional HbA1c, LDL, and blood pressure readings 6 months after the last face-to-face visit.

2.1.3. exact location where data will be collected,

CMC VAMC and the CMCVAMC Annex (for in person, phone and chart derived data).

2.1.4. exact location where data entry will take place.

All data entry will occur either at the CMC VAMC or the CMC VAMC Annex. Paper files for the study will be housed at the CMC VAMC annex in locked filing cabinets. Data will be stored on VA servers behind the VA fire wall. .

2.1.5. the "title" of individual(s) collecting the data and analyzing the data, e.g. principal investigator, research coordinator.

A Project manager and/or research coordinators will be collecting data and entering it into the database. The Principal investigator and a master's level programmer will be analyzing the data. Identifiable data will not be removed from CMC VAMC servers. Access to identifiable data will be limited to those who require access including the project manager and research coordinators, principal investigator, and the programmer.

2.2. Provide a time line for each aspect of the study.

Baseline: Obtain consent, complete baseline survey, randomize, obtain blood.
For arm 2 (and 4) also: Identify mentor, obtain consent from mentor, complete mentor baseline survey, train mentor, obtain blood from mentor, have mentor start calling mentee.

6 months: Complete follow up survey, interviews, and blood for mentors and mentees.
For arm 2 also: Train arm 2 participants to be mentors and match them to mentees (participants in arm 3).

12 months: Complete follow up survey, interviews, and obtain blood from arms 1, 2, 3, and 4.

18 months: Complete follow up survey, interviews, and obtain blood from arms 2, and 4.

18 months-24 months: Monitor electronic health record for arm 1, 2, 3, and 4.

Ongoing: Call all mentors every month to reinforce training and determine mentor-mentee call frequency. Call all mentees at 1 and 3 months to assess hypoglycemic symptoms.

2.3. Chart/Records/Data Review (retrospective and/or prospective)

2.3.1. Provide the planned or approximate number of charts/records/data to be accessed

2.3.1.1. CMCVAMC

Given our previous experience we anticipate having to screen between 1,600-2,000 charts to identify 492 poorly controlled diabetic veterans and 206 mentors who will be eligible and willing to participate.

Similarly we expect to screen 1000 additional charts to identify 206 mentors who are eligible and willing to participate, as well as mentors to replace those who withdraw.

2.3.1.2. Other site

2.3.2. Does this protocol employ an Honest Broker? ☐YES **X**NO

2.3.2.1. If yes, provide name of individual.

2.3.2.2. If no, explain who will access the charts/records.

2.3.2.3. Describe from what database charts/records/data will be accessed.

3. **Future Use of Data and Re-Contact**, if applicable. N/A
   3.1. If any of the participant's data are going to be retained after the study for future research, the following information must be provided to the participant:
      3.1.1. Where will the data be stored?
      3.1.2. Who will have access to the data?
   3.2. If the subject is going to be re-contacted in the future about participating in future research, this must be specified. Describe the circumstances under which the participant would be re-contacted whether within the VA or outside the VA.
      3.2.1. If subjects will receive aggregate study results at the end of the study, the informed consent document must contain this information.

4. **Specimen Collection**
   4.1. Give the source of all specimens and whether they were collected for research, treatment or diagnosis.

Blood will be drawn to assess HbA1c and direct LDL on three-four different occasions for research purposes. Treatment decisions will not be based on results by the research team.

4.2. State where specimens will be stored, secured and when discarded.

Specimens will be drawn by the CMC VA Medical Center lab and disposed of appropriately. We will not be storing blood for future use.

4.3. Explain how destruction of samples will be substantiated.

The CMC VA Medical Center lab will handle blood samples in the same way they handle samples for routine clinical care and subject to the oversight of routine clinical care.

N. **Genetic Testing, if applicable** N/A

1. Explain if the study is looking for an association between a genetic marker and a specific disease or condition, but at this point it is not clear if the genetic marker has predictive value.

   1.1. The uncertainty regarding the predictive value of the genetic marker is such that studies in this category will not involve participant counseling.
   1.2. Describe if the study is based on the premise that a link between a genetic marker and a specific disease or condition is such that the marker is clinically useful in predicting the development of that specific disease or condition.
   1.3. Will the subject be notified of the results and the provision for genetic counseling?
   ☐ Yes ☐ No ☐ N/A
      1.3.1. If yes, explain further.
   1.4. If biological specimens are used in this protocol, please respond to the following questions by checking the appropriate box:

	YES	NO	N/A
a. Does the project involve genetic testing?	☐	☐	☐
b. Will specimens be kept for future, unspecified use?	☐	☐	☐
c. Will samples be made anonymous to maintain confidentiality? <u>*(Instructions: Note: If there is a link, it is not anonymous. Coding is not anonymous.*</u>	☐	☐	☐

d. Will specimens be destroyed after the project-specific use is completed?	☐	☐	☐
e. Will specimens be sold in the future?	☐	☐	☐
f. Will subjects be paid for their specimens now or in the future?	☐	☐	☐
g. Will subjects be informed of the results of the specimen testing?	☐	☐	☐
h. Are there any implications for family members based on specimen testing results? (If yes, they may be participants.)	☐	☐	☐
i. Will subjects be informed of results obtained from their DNA?	☐	☐	☐

1.5. Will specimens be de-identified? ☐YES ☐NO ☐ N/A
 1.5.1. If yes, please describe the procedures to be used.
 1.5.2. Include at what point in the process the specimens will be de-identified.
1.6. Describe what measures will be taken to minimize the following risks from breaches of confidentiality and privacy resulting from participating in **THIS aspect** of the research project:
 1.6.1. physical
 1.6.2. psychological
 1.6.3. financial
 1.6.4. social
 1.6.5. legal harm

O. **Banking of Collected Specimens**
 1. **Will collected specimens be banked?** ☐YES XNO ☐N/A
 1.1. *IF BANKING SPECIMENS, IT MUST BE AT AN APPROVED VA REPOSITORY.* (*For additional information, refer to VHA Handbook 1200.12, Use of Data and Data Repositories in VHA Research - March 9, 2009.)*
 1.2. If yes, specify the location where specimens will be banked.
 1.2.1. If the location is a non-VA site, has the mandatory approval from the Chief Officer of Research and Development (CRADO) been obtained through submission of a tissue banking application (VA Form 10-0436 - Off-site Application for an Off-site Tissue Banking Waiver)? ☐YES ☐NO ☐N/A
 1.2.2. If applicable, attach a copy of the VA Form 10-0436
 1.3. Explain how destruction of banked samples will be substantiated.

P. **Subject Recruitment** (characteristics of the study population)
 1. **Provide the planned or targeted enrollment at:**
 1.1. CMCVAMC - 492 poorly controlled diabetic veterans and 206 mentors, as well as mentors to replace those who withdraw.
 1.2. Other sites -
 1.3. Not applicable; chart review or use of previously collected data - ☐

 2. **Screening and/or Eligibility Requirements**
 2.1. Describe and provide justification for:
 2.1.1. Inclusion criteria

All participants and mentors will be type 2 diabetics. To be eligible they must have developed diabetes after the age of 30. To ensure that we are targeting those most likely to benefit from improving control we enroll diabetics with persistently poor DM control. Persistently poor DM control will be defined as having an HbA1c > 8% on 2 different occasions in the course of 24 months, with at least 1 measure within 3 months of enrollment. Mentors for arm 2 will have previously been in poor control and are now in good control, defined as having an HbA1c of > 8% in the past 3 years and an

HbA1c ≤ 7.5% within 3 months of enrollment. Also, to ensure that we are targeting those most likely to benefit from improving control, participants will need to be between the ages of 30-75.

2.1.2. Exclusion criteria

We will have minimal exclusion criteria. Patients will be excluded if they do not speak English or are unable to understand the consents as exhibited by failing the mini quiz. Patients will also be excluded if they have a severe speech impediment of any kind since they need to be able to converse with a mentor or mentee over the telephone. Patients over the age of 75 will also be excluded since it is unclear if they will derive the same benefit from getting their HbA1c in control.

2.2. List all screening and/or eligibility requirements.

We will electronically create a list of CMCVAMC patients with at least two diagnosis codes for diabetes (ICD-10 code 250) and least two laboratory tests in for an HbA1c. We will exclude people if their current age is ≤ 30 and > 75 years of age. We will then review the list and determine who qualifies as a potential mentee and a potential mentor. Letters will be sent to potential participants letting them know that they may be eligible for a study with a brief description of the study and give them a number if they would like to learn more or to request no further contact. We will also notify potential participants that if we do not hear from them we will give them a call in the following weeks.

Potential mentors will be identified and contacted in a similar manner.

2.3. Explain any special test or evaluations potential subjects may have to undergo before they are actually determined to be eligible for the study.

When patients are contacted over the phone we will give them an additional description of the study and for those interested we will ask them at what age they developed diabetes. Those who indicate they developed diabetes before the age of 30 will be excluded from participating. The same will be true for when we contact potential mentors. We will also ask them about how comfortable they are with speaking English. Those who indicate discomfort with speaking English or are unintelligible over the phone will also be excluded from participating.

2.4. Not Applicable; subjects not recruited; chart review. ☐

3. **If applicable, indicate what populations will be targeted for recruitment as participants. Check all that apply.**

Males	X
Females	X
Inpatients	☐
Outpatients	X
VA Employees	☐
Non-English Speaking**	☐
Veteran Family members***	☐
Non-Veterans***	☐
Other (Specify)	☐
Not Applicable, chart review	☐

3.1. **For non-English speaking subjects - If an investigator proposes to use a participant population that does not speak or read English, a copy of the translated document, as

well as the English version, needs to be forwarded to the IRB for approval. Translator certification is also required.

3.2. ***If non-veterans will be recruited for this study, explain why sufficient veterans are not available to participate in the project [VHA Handbook 1200.5, paragraph 16a]. Veteran's spouses/partners, caregivers, etc. are considered non-veterans for the purposes of this study.

3.3. ***Has approval to recruit non-veterans been received from the ACOS/R&D and Medical Center Director?

3.3.1. X Not Applicable

3.3.2. ☐ Pending *(Non-veteran forms should be used. IRB office will obtain approval from ACOS/R&D and Medical Center Director.)*

4. **Does this project target a specific race or ethnic group as participants?** ☐YES X NO
If yes, check all that apply.

**Race**	
American Indian or Alaskan Native	☐
Asian	☐
Black or African American	☐
Native Hawaiian or other Pacific Islander	☐
Black, not of Hispanic origin	☐
White, not of Hispanic origin	☐
Other	☐

**Ethnicity**	
Hispanic or Latino	☐
Not Hispanic or Latino	☐
Other	☐

4.1. Provide justification why this/these group(s) was/were chosen.

5. **What is the age range of participants?** Check all that apply.

Children (Under 18) Requires Waiver from CRADO (VHA Directive 2001-028, Research Involving Children)	☐
Young Adults (18-21)	☐
Adults (22-65)	X
Seniors (Over 65)	X
Over 89	☐
Not Applicable, chart review	☐

6. **Are there specific reasons why certain populations (i.e., age, gender or ethnic groups) are excluded as participants?** ☒YES X NO ☐N/A

6.1. If yes, specify reasons. We excluded people in the age groups younger than 30 years and older than 75 years of age. Patients under the age of 30 were excluded to ensure we are identifying a population with type 2 diabetes, and patients over the age of 75 will also be excluded since it is unclear if they will derive the same benefit from getting their HbA1c in control.

7. **Does the project require enrollment of the following classes of participants?**

	YES	NO
a. Employees	☐	X
b. Individuals with impaired decision making capability	☐	X
c. Pregnant women	☐	X
d. Economically and/or educationally disadvantaged persons	☐	X
e. Prisoners	☐	X
f. Illiterate, limited, or no English language proficiency	☐	X
g. Terminally ill patients	☐	X

While the study does not require the enrollment of these groups we anticipate that we will enroll economically and/or educationally disadvantaged people as well as people who may have limited health literacy. All study materials including consent forms will be read to participants. Only those who fail the mini-quiz and thus seem not to comprehend the consent process will be excluded.

7.1. If applicable, what is the justification for including any of the above classes of participants in the project?
7.2. If the project requires enrolling any of the above classes of participants describe any project-specific measures or special considerations, steps, or safeguards to ensure that these individuals are adequately protected.

8. **Describe the exact plan how subjects will be identified and recruited for the study.**

8.1. Discuss methods, e.g., referrals from physician offices, clinics, programs, or through advertisements and brochures.

We will electronically create a list of CMCVAMC patients with at least two diagnosis codes for diabetes (ICD-9 code 250 and ICD 10 codes starting with E11) and at least two laboratory tests in for an HbA1c. We will exclude people if their current age is ≤ 30 and > 75 years of age. We will then review the list and determine who qualifies as a potential mentee and a potential mentor based on past HbA1c values. This will be an ongoing process.

Letters will be sent to potential participants letting them know that they may be eligible for a study with a brief description of the study and give them a number of the project manager who they can call if they would like to learn more or to request no further contact. We will also notify potential participants that if we do not hear from them we will give them a call in the following weeks. Potential mentors will be identified and contacted in a similar manner.

When patients are contacted over the phone we will give them additional description of the study and for those interested we will ask them at what age they developed diabetes. Those who indicate they developed diabetes before the age of 30 will be excluded from participating. The same will be true for when we contact potential mentors. We will also ask them about how comfortable they are with speaking English. Those who indicate discomfort with speaking English or are unintelligible over the phone will also be excluded from participating.

Those interested in participating in the study will be invited into the CMC VAMC for a face to face meeting with the study team. At that time if still interested participants and mentors will complete the consent process in person including completing a mini-quiz about the process. Those who fail the mini-quiz will be excluded from the study at that time; however, they will be reimbursed the $50 for coming in for an in person visit. We anticipate almost everyone who comes in for a face to face visit will be entered into the study.

9.

9.1. If using a clinic, be specific about who will identify the potential subject and how that information will be transmitted to the research staff.
9.2. If snowball method will be used, discuss the process and how the first individuals will be recruited.
9.3. Describe how information will be disseminated to subjects, e.g. handouts, brochures, flyers and advertisements *(include all recruitment materials with this submission)*.

10. **Informed Consent**

10.1. Informed Consent will not be sought. ☐
10.2. Written informed consent from participants (VA Form 10-1086 is attached). **X**

10.3. Written informed consent from participants' legally authorized representative (LAR) as required by VA policy and/or applicable state laws (VA Form 10-1086 is attached). ☐
10.4. Request Waiver of Documentation of Informed Consent ☐
10.5. List the **title** of the key personnel involved in the following activities:

10.5.1. Person Obtaining Consent

10.5.1.1. Provide the title(s) of individual(s) Program Specialist
10.5.1.2. Type of training received to perform this process CPRS training, CITI training, HIPPA training, and scanner training.

10.5.2. Pre-Recruitment Screening (the use of medical records and other data bases to determine populations and individuals eligible for the study), **Data Base Programmer and Project Manager or Program Specialist**

10.5.3. **Recruitment Process (**the process in which individuals are contacted and first introduced to the study and to the possibility of participating as subjects), **Project Manager or Program Specialist**

10.5.4. **Informed Consent Process** (the process by which recruited subjects are fully informed about participating in the study and then formally give their voluntary consent for participating), **Project Manager or Program Specialist**

10.5.5. **Screening of Recruited Subjects** (those activities in the protocol in which a final determination of eligibility of prospective subjects is made during the early phases of the study, using laboratory data, inclusion and exclusion criteria, and other person-specific information), **Project Manager or Program Specialist**

10.5.6. Include the breakdown of each individual's responsibilities:

10.5.6.1. Principal Investigator, .**Judith A.Long, M.D. will be responsible for the oversight of this entire study**
10.5.6.2. Co-Principal Investigator, N/A
10.5.6.3. The Project Manager Jennifer Gutierrez or Research Coordinator, Tanisha Dicks or other IRB approved research staff will be responsible for the IRB administration, screening, the recruitment and consent, data collection including measuring BMI, blood pressure, and interviews (over the phone and in-person). They will also be responsible for training the mentors and mentee-mentor coordination.
10.5.6.4. Additional research staff by title, **The Project Manager Jennifer Gutierrez or Research Coordinator, Tanisha Dicks will be responsible for IRB administration.**

10.6. Will informed consent be obtained from potential subjects prior to determining eligibility?
☐YES X NO ☐N/A

10.6.1. If no, provide justification and a HIPAA Waiver of Individual Authorization for Disclosure of Protected Health Information.

To make the recruitment process efficient we will need to screen for patients with diabetes, in the correct age range, with the lab results as described above. Please see the attached HIPAA Waiver of Individual Authorization for Disclosure of Protected Health Information for approval of this activity.

10.7. Define when a subject is enrolled into the study, e.g. after the subject signs the informed consent or after randomized to treatment.

After the informed consent is complete the patient will be enrolled in the study.

10.8. Describe:

10.8.1. The process when informed consent will be obtained and protecting patients' privacy.

Informed consent will be obtained at the CMC VA Medical Center in a semi-private room with cubicles. This room usually has only one person working in it at a time and thus affords audio privacy. All study materials including informed consent forms will be read to the participant.

10.8.2. Any waiting period between informing the prospective participant and obtaining consent. **No**

10.8.3. Steps taken to minimize the possibility of coercion or undue influence.

The consent process will explain how participation is voluntary and will not affect the care or services the patient is eligible from the Philadelphia VA Medical System. Participants will be told they can withdraw from the study at any time. Participants will only receive a reimbursement to defray the costs of travel and cover the participant's time.

10.9. Provide the language

10.9.1. used by those obtaining consent Please see attached script.

9.9.2 understood by the prospective participant or the legally authorized representative Please see attached script.

10.10. Provide location where informed consent will be obtained. **The CMC VA Medical Center**

11. **Waiver or Alteration of Informed Consent Requirements/Waiver of Requirement to Obtain Documentation of Informed Consent**

11.1. Are you requesting a waiver or alteration of informed consent? *(Check all that apply)*

11.1.1. No **X**

11.1.2. Yes; provide justification. ☐

11.1.3. Yes; for recruitment purposes only. ☐

11.1.3.1. An IRB may approve a consent procedure which **<u>does not include, or which alters</u>**, some or all of the elements of informed consent set forth in this section, or waive the requirements to obtain informed consent provided the IRB finds and documents that:

- ☐ 1. The research involves no more than minimal risk to the subjects;
- ☐ 2. The waiver or alteration will not adversely affect the rights and welfare of the subjects;
- ☐ 3. The research could not practicably be carried out without the waiver or alteration; and
- ☐ 4. Whenever appropriate, the subjects will be provided with additional pertinent information after participation
- ☐ 5. The research or demonstration project is to be conducted by or subject to the approval of state or local government officials and is designed to study, evaluate, or otherwise examine:
  a. Public benefit or service programs;
  b. Procedures for obtaining benefits or services under those programs;
  c. Possible changes in or alternatives to those programs or procedures; or

d. Possible changes in methods or levels of payment for benefits or services under those programs.

11.2. **Are you requesting a waiver to obtain documentation of informed consent?**

11.2.1. No **X**

11.2.2. Yes; provide justification. ☐

11.2.2.1. An IRB may **waive the requirement for the investigator to obtain a signed consent** form for some or all subjects if it finds either:

- ☐ 1. That the only record linking the subject and the research would be the consent document and the principal risk would be potential harm resulting from a breach of confidentiality. Each subject will be asked whether the subject wants documentation linking the subject with the research, and the subject's wishes will govern; or
- ☐ 2. That the research presents no more than minimal risk of harm to subjects and involves no procedures for which written consent is normally required outside of the research context.
- *NOTE: In cases in which the documentation requirement is waived, the IRB may require the investigator to provide subjects with a written statement regarding the research.*

Q. **Compensation** *(The amount of compensation may not constitute an undue inducement to participate in the research.)*

1. Summarize any financial compensation that will be offered to subjects.

   All participants will receive $50 for each in person visit. Mentors will also receive an additional $20 per month if they call their mentee 4 times per week. Mentors and mentees will receive an additional $40 for each qualitative interview completed.

2. Provide the schedule for compensation. **Baseline, 6 months, 12 months, and for arm 2 and 4 18 months.**
   2.1. Per study visit or session. $50
   2.2. Total amount for entire participation. **Arm 1 $150, Arm 2 up to $460, Arm 3 up to $270, Arm 4 up to $320.**
3. Explain how compensation will be provided via cash, voucher, gift card, etc. **Participants will be given vouchers which they can then redeem for cash from the CMC VA cashier.**
4. If financial compensation will be prorated, explain the process. **N/A**
5. Not Applicable - ☐

R. **Withdrawal/Early Withdrawal**

1. Describe how and when a subject may withdrawal from the study. **Subjects may withdraw at any time. Any request may either be expressed verbally or in writing.**

2. Provide procedures for the orderly termination of participation by the participant and if any consequences would result from early withdrawal from the study.

   There will be no consequence of early withdrawal. After submitting a request to withdraw or written or verbally the study participation will be terminated. If a mentor withdraws before 4 months, his or her mentee will be re-matched to another mentor.

3. Explain if survival data is required. If so, clarify how data will be obtained. **N/A**
4. Not Applicable; subjects not recruited; chart review.

S. **Risk/Benefit Assessment**

1. **Potential Study Risks**

1.1. Describe and assess all of the following risks that may be associated with the research:

1.1.1. Physical **Minimal**
1.1.2. Psychological **Minimal**
1.1.3. Social **Minimal**
1.1.4. Economic **Minimal**
1.1.5. Monetary **Minimal**
1.1.6. Legal **Minimal**
1.1.7. Loss of confidentiality **Minimal**
1.1.8. Assess the likelihood and seriousness of such risks. **Minimal**
1.1.9. Other

1.2. Specify what steps will be taken to minimize these risks.

Patients will need to complete a mini-quiz after completing the informed consent to assure the researchers they understand the consent procedure. Those who fail the mini-quiz will be given $50 for the in person visit but not enrolled in the study. All participants will be notified that participation is voluntary and they can terminate participation at any time. They will also be notified that their care will not be affected by their participation.

The greatest risk to patients will be loss of confidentiality. Mentors and mentees will be informed that their conversations are confidential and should not be shared. Mentees will need to provide a working phone number which will be provided to the mentor. Mentees will be informed of this process up-front.

Tracking data with identifiable information will be kept in a different data base than the analytic data base. All databases will be password protected and reside on VA servers. Neither data base will be removed from the VA server and will be behind VA firewalls. Paper files will be kept in locked filing cabinets and will not leave the CMC VAMC or Annex. Papers will be kept in locked files when not being actively used. All participants will be given a study ID number and this will be the only link between the analytic files and the tracking data base.

1.3. If methods of research create potential risks, describe other methods, if any, that were considered and why they will not be used. **N/A**

1.4. If chart review, breach of confidentiality is always a concern. Specify what steps will be taken to minimize these risks.

Charts will be reviewed to determine if a potential subject may be eligible for the study. Only IRB approved study personnel will access charts from VA computers during work hours. Abstraction will be limited to determine eligibility only.

Chart will also be reviewed 6 months after the last in-person visit has taken place (and for those lost to follow-up should have taken place). Minimal data will be abstract from the chart -- additional HbA1c, LDLs, and BP measurements from the time of entry into the study to the day of the chart abstraction. Only those with IRB approval will abstract this data and it will only be done on the VA premises. Data written on paper abstraction tools will be entered into the analytic database house behind the VA firewall. Paper documents will be kept on site and in locked filing cabinets in the CMC VAMC Annex.

2. **Potential Study Benefits**

2.1. Assess the potential benefits to be gained by the individual subject, as well as benefits that may accrue to society in general as a result of the planned work.

In our pilot study, poorly controlled diabetics who received mentoring on average improved their HbA1c by 1%. This is a large and clinically significant improvement. If improvements persist then the participant may reduce his or her risk of future complications from diabetes. This study in part will help us understand better if risks persist. This study has the potential also to add to our knowledge about how to better to support diabetics in general with behavioral change.

2.2. If the subject does not receive any direct benefit, then it must be stated here and in the consent form.

While we hope that subject will receive benefit it is likely that some participants will not benefit especially those who are randomized to usual care.

3. **Alternate Procedures**
   3.1 Describe the alternatives available to the subject outside the research context. **N/A**
   3.2 If none, state that the alternative is not to take part in this research study at all. **The only alternative is to not take part in the research study.**

T. **Data and Safety Monitoring Board (DSMB) or Data Monitoring Committee (DMC)** (All Phase III studies are required to have a DSMB. *However, the IRB has the right to require a DSMB with any study.*)

1. **Will an independent DSMB or DMC oversee the project?** ☒YES ☐NO ☐N/A
   2.1. If yes, please provide contact information for the DSMB or DMC or Coordinating Center Representative and attach a copy of the charter.
   Name: Joshua Metlay MD   Phone Number: (610) 519-9168

   Title: DSMB Chair   E-mail: jmetlay@exchange.upenn.edu

2. **If a DSMB or DMC will not monitor this study, who will monitor this study? Check all that apply.**
   X Principal Investigator
   ☐ Sponsor
   ☐ VA Cooperative Studies Program
   ☐ Safety monitoring committee

U. **Data Monitoring** (*Monitoring plans describe how a monitor, independent of the study team, regularly inspects study records to ensure the study is adhering to the study protocol and applicable research regulations and CMCVAMC requirements. Monitoring plans do not necessarily require the use of an independent Data and Safety Monitoring Board (DSMB). Such independent boards are usually reserved for high-risk phase I studies, or large, multi-center phase III trials. Federally funded studies may require the use of an independent DSMB.*)

1. **Describe the data monitoring plan.** (All protocols must have a data monitoring plan appropriate for the potential risks and the complexity of the study.)

Hypoglycemic events will be monitored at 1, 3 and 6 months. All serious adverse events will be reviewed by the PI and reported to the IRB as well as the DSMB. We do not expect events to differ by arm based on our prior research.

Data not directly entered will be double entered to ensure fidelity. Paper consents will be reviewed routinely for completeness. All files will be regularly maintained and available for inspection upon request.

2. **Describe how protocol deviations, adverse events, serious adverse events, breaches of confidentiality, unanticipated adverse device effect (UADE), and unanticipated or unexpected problems will be reported to the CMCVAMC IRB and sponsor.** *(Refer to the CMCVAMC IRB Standard Operating Procedure (SOP) Manual for reporting guidelines.)* **Breaches of confidentiality will reported within 24 hours of our awareness of said breach.**
   2.1. Describe the management of information obtain that might be relevant to participant protections such as:
      2.1.1. Unanticipated problems involving risks to subjects or others

This is a low risk study. However, if unanticipated problems arise the study team will review them and report them immediately to the CMCVAMC IRB. If there are serious concerns about risks to subjects or others the study will be halted while an external group reviews the study to determine the risks.

2.1.2. Interim results

We are not planning an interim analysis.

2.1.3. Protocol modifications

If we desire to modify the protocol we will submit a modification to the IRB and not act on the modification until it has been approved.

3. **If applicable, define the plan for subjects if research shows results such as:**
   3.1. Depression **We will only use a depression screening tool and not assessing for depression. If the participant is willing and they screen positive for depression we will notify the participant's primary care provider.**
   3.2. Suicide **Will not be assessed**
   3.3. Abuse **Will not be assessed**

4. **Statistical Analysis**
   4.1. Include statistical power calculations and the assumptions made in making these calculations.

We will randomize 72 mentees from stage 1 to each arm of stage 2. To achieve 80% power to detect a 0.8 unit change in HbA1c, with standard deviation of 1.6, a sample of 64 patients per arm would be required. But to protect against possible attrition, we will inflate that by 10% and enroll 72 participants into each arm. Literature and guidelines suggest that an average decrease of 1 unit in HbA1c would provide a clinically meaningful improvement in health. We chose a 0.8 unit change to be conservative. We base our standard deviation of 1.6 on our pilot study and other studies of low control DM populations.

For stage 2 we will also randomize poorly controlled diabetics into mentoring or usual care arms. We will need 72 in the usual care arm (to be mentored by the 72 stage 1 mentees who become mentors). Since usual care for stage 2 can be combined with usual care for stage 1, there is no need for a 1:1 randomization. Initially, a 1:1 randomization was used, but after evaluation we

believe we can now switch to a 2:1 randomization to avoid recruiting more usual care patients than necessary. With this change we will enroll approximately 56 patients to usual care in stage 2.

In order to assure 72 in each arm for stage 2, we will need a total of 144 mentees in stage 1. Again, to protect against possible attrition, we inflate that by 16% and enroll 171 mentees in stage 1. A corresponding number of mentors (171) will be recruited for stage 1. There will be no randomization of mentors in stage 1. Since usual care for stage 1 can be combined with usual care for stage 2, there is no need for a 1:1 randomization. Initially, a 1:1 randomization was used, but after evaluation we believe we can switch to a 2:1 randomization to avoid recruiting more usual care patients than necessary. With this change we will enroll approximately 139 patients to usual care in stage 1.

The combined number of poorly controlled diabetic patients receiving usual care will be approximately 195. The number of poorly controlled diabetic patients receiving peer mentoring will be 232 (171 in stage 1 and 72 in stage 2). The number of mentors in stage 1 will be 171, and the number of mentors in stage 2 will be 72 (from among those who received mentoring in stage 1). The total number of patients in the study will be 594.

A recent analysis of clinical data from the CMC VAMC and its surrounding CBOCs indicate that in the last year there were 1,248 patients who would have qualified to be mentees and 1,810 who would have qualified to be mentors.

4.2. Define plans for data and statistical analysis, including key elements of the statistical plan, stopping rules and endpoints.

For Stage 1: We will test these primary hypotheses using an unadjusted intent-to-treat analyses, i.e., with adjustment for only baseline HbA1c. The primary tests will assess the significance of the coefficients associated with the treatment assignment in a longitudinal linear model of the repeated HbA1c as a function of treatment, where the active group (peer mentoring) is compared to the control group via an indicator variable. Standard t-tests will determine whether a significant difference in Hb1Ac improvement occurred between the treatment arm compared to the control arm. To account for the fact that we are doing multiple tests, we will use the Bonferroni method and consider a treatment to be significantly different than the control if the p-value is less than 0.05/2=0.025.

Stage 2 Analysis:

<u>H2. Compared to usual care, veterans who receive peer mentoring from former mentees will have improved glucose control as measured by HbA_{1c}, BP, LDL levels, DM quality of life, and depression scores:</u> We will examine the effectiveness of the intervention at 6 months and further examine sustainability at 12 and 18 months. 18 month assessments will only be of HbA_{1c}, BP, and LDL levels as derived from the EMR. We will test these primary hypotheses using an intent-to-treat (ITT) analyses, i.e., including all patients who completed a baseline assessment, as randomized. We will use linear and random effects longitudinal models to perform the ITT comparisons at each of the follow-up visits. Our primary outcomes will be HbA_{1c}, BP, LDL levels, DM quality of life, and depression scores. Our models will include a random effect for patient. The models will include the following fixed effects: a main effect for follow-up visit (with baseline visit as the reference), intervention group, and their interactions. Tests of the interaction terms will be used to determine the ITT difference between groups with respect to change from baseline. To examine whether veterans who received peer mentoring from former mentees have improved glucose control, BP, LDL levels, DM quality of life, and depression scores compared to veterans in the usual care group at 6 months, compared to baseline, we will implement these random effects models with mentee HbA_{1c}, BP, LDL, DM quality of life score, or depression score as the dependent variable. The ITT test for effectiveness will be based on HbA_{1c} and will be two-sided, with

alpha=0.05 and will be based on the magnitude and statistical significance of the time*intervention interaction term at 6 months. To examine sustainability of improvement in glucose control, BP, LDL levels, DM quality of life, and depression scores we will use the same test, but will examine the intervention group*12 month time interaction term. To examine sustainability of improvement in glucose control, BP, and LDL levels at 18 months we will again use the same test, but will examine the intervention group*18 month interaction term.

We will check model fit by inspecting residual plots, and will assess improvement in fit with different correlation structures for the random effects. While we do not anticipate confounding in our ITT estimates because of the stratified and blocked randomization of patients, confounding due to imbalance in patient characteristics may still occur by chance. Tests for evidence of confounding will be performed by comparing baseline characteristics, between control and intervention group. Confounders will be identified as variables that are significant at the 0.20 level and we will include these as adjustment variables (fixed effects) in our analyses. These multivariable models will reduce the impact of potential bias due to confounding, as well as improve the statistical power by reducing the amount of unexplained variation in the outcome variable.

Extensive efforts will be made to collect complete information on each participant enrolled in the study. While we do not expect to be confronted with nonignorable missing data, we will nevertheless assess the sensitivity of treatment effect estimates to missing outcome data in a number of ways. We will compare the number of visits completed by participants in each arm as an additional secondary outcome, using the chi-square test. We will also compare observed characteristics of subjects with complete follow-up to those with missing data and will attempt to ascertain the reasons for missing data in subjects with incomplete follow-up.

H3: Compared to past mentees who are not randomized to becoming a mentor, past mentees randomized to becoming a mentor will have better glucose control, BP, LDL levels, DM quality of life, and depression scores: To examine whether past mentees who become mentors have better glucose control, BP, LDL levels, DM quality of life, and depression scores than past mentees who do not become mentors, the dependent variable in this analysis will be past mentee HbA_{1c}, BP, LDL level, DM quality of life score, and depression score and the intervention group will be the randomization group for former mentees. As we described above, tests of the interaction terms for each follow-up time (i.e. 6, 12 months, and 18 months) with intervention group will be used to determine the ITT difference between groups with respect to change from baseline HbA_{1c}, BP, LDL level, at each follow-up time and DM quality of life score, and depression score at 6 and 12 months.

Testing of Secondary Quantitative Hypotheses:

H4. In those randomized to being a mentee, explore mentor characteristics associated with improved outcomes. Predictors to be evaluated include past mentoring dose of the current mentor, the mentor's past change in HbA_{1c}, the mentor's starting HbA_{1c}, current mentoring dose provided by the mentor, the mentee's evaluation of the mentor, and the mentor's depression score at baseline. Based on the literature and our pilot studies, we have opted to use past mentees as mentors regardless of a. time passed since they were a mentee, b. the amount improvement in HbA_{1c} when they were a mentee, or c. their starting HbA_{1c} in this study. We will evaluate if any of these mentor variables are associated with mentee change in HbA_{1c}, BP, LDL level, DM quality of life score, and depression score at 6 and 12 months and HbA_{1c}, BP, and LDL level at 18 months.

Assessment of Hypoglycemia: In order to assess the relative frequency of minor hypoglycemic symptoms we will summarize the number of participants in each study arm reporting 0, 1-3, or >3 minor hypoglycemic symptoms per month. In order to compare average rates per person per month, using all available data, we will fit a generalized linear mixed model with random subject intercepts and test for differences between treatment groups. Based on our previous study we expect very few major hypoglycemic events, we will compare differences by arm using a Fisher's exact test. If requested by the

Data Safety Monitoring Board (DSMB – see Section E.5 in Human Subjects), preliminary analyses of minor and major hypoglycemic events will be completed before the completion of the study.

Qualitative Analyses:

Process Evaluation: To facilitate later deployment of results and to improve the design of future interventions, we will engage in a qualitative process evaluation throughout the study to learn why some study participants succeed in changing behavior and others do not, and what elements of the approach were acceptable to participants.[115] In particular, we are interested in learning about the mentor-mentee relationship. All interviews will be audio-taped, transcribed, and content-analyzed. We will use the framework of adult learning theory to analyze the results. We will look for evidence of the following from successful mentor mentee pairs: 1. the mentee expressed a perceived a need to change, 2. the process was relevant to the mentee's needs, 3. the process motivated the mentee to change, 4. the mentee felt the mentor listened to the mentee and supported the mentee's identified goals, 5. The mentor was motivated to be a mentor, 6. the mentor tailored their approach to the needs of the mentee, and 6. the mentor fostered and enhanced the mentee's sense of self-efficacy regarding diabetic health behaviors. However, while we use the lens of adult learning theory to guide the analysis we will keep an open mind to the potential that this framework does not adequately characterize all aspects of successful pairs and will look for common themes that might lie outside of this framework. Following this process of careful reading and reflecting upon the interview transcripts and discussing them among ourselves will provide an opportunity to understand the mechanisms by which peer mentoring operates and identify themes that emerge from the data.

After extensive discussion of the transcripts and identification of important themes, we will generate a Working Coding Scheme (WCS) based on the theoretical model and refine it through content evaluation of the first few interviews. Once we have developed an initial WCS, Dr. Long and the project manager will independently recode the content of the already coded interviews, and through an iterative process with Dr. Shea, discrepancies will be resolved until a final coding scheme is developed. This similar process of double coding with resolution of disagreements will then be applied to coding all the transcripts.

The final WCS will be used as an analytic tool for identifying patterns in the data, answering conceptual research questions, and generating new hypotheses grounded in the data. For analysis of transcripts, we will use NVivo software for qualitative data analysis. We will use the software to develop counts of codes stratified by change in HbA_{1c} as well as by the mentor's past experience with receiving mentoring (optimal/suboptimal) and the mentor's past success with improving their HbA_{1c}. We have used this analytic approach previously and found it a meaningful way by which to identify themes and understand the emphasis placed on the theme by the populations as a whole. These analyses will be used to refine the conceptual model and expand our understanding of how peer mentoring functions. In addition, comparisons will be made to determine if there is thematic consistency between mentors and mentees.

By collecting qualitative data sequentially in conjunction with the quantitative study we will be able to interpret and contextualize our quantitative findings and inform the implementation of peer mentoring programs.

V. **Privacy and Confidentiality** *(Privacy refers to persons and to their interest in controlling the access of others to themselves.) (Confidentiality refers to protecting information from unauthorized disclosure or intelligible interception.) (Investigator should contact the Privacy Officer for additional details.)*

1. **Indicate the type of data that will be received by the Principal Investigator. Check all that apply.**

1.1. ☐ De-identified – Without any identifiers that could link the data to a specific participant. (Contact Privacy Officer for assistance. _If data is coded, it is not considered de-identified.)_

1.2. X Identified – Linked to a specific participant by identifiers sufficient to identify participants. (See HIPAA and Common Rule Criteria for list of identifiers.)

1.3. ☐ Coded – Linked to a specific subject by a code rather than a direct identifier. If coded is checked, specify:

1.3.1 Explain who will maintain the link or code.

1.3.2 Describe who will have access to the link or code.

1.3.3 Provide exact details for how the data is coded.

1.3.4

2. **Does the project require the use of existing Protected Health Information (PHI) from a database, medical records, or research records? X** YES ☐NO ☐N/A

2.1. If yes,

2.1.1. Specify the source of the existing PHI **VISN 4 VA Electronic Records**

2.1.2. Indicate the specific data elements/identifiers (e.g., name, address, phone numbers, etc.) on the below table. See below:

For contact purposes and tracking: name, address, phone numbers, date of birth, and social security number.
For determining eligibility: diagnosis codes of diabetes, HbA1c levels, and date of birth.

Please see the attached HIPPA waiver requesting permission to access this data.

2.2. If the study uses an existing database/data warehouse,

2.2.1. Provide a description of the database/data warehouse. To identify potential eligible participants we will pull names from the VISN 4 data warehouse.

2.2.2. Make clear who is responsible for maintaining it. This warehouse is maintained by the VISN.

2.2.3. Cite any relevant Standard Operating Procedures (SOP) for the database/data warehouse. **N/A**

2.2.4. Provide a copy of the SOP.

3. Will PHI be collected prior to obtaining informed consent? **X** YES ☐NO ☐N/A

3.1.1. If yes, complete and provide a HIPAA Waiver of Individual Authorization for Disclosure of Protected Health Information with this submission.

4. HIPAA Identifiers - Indicate the PHI that will be collected from project participants directly or indirectly.

4.1. X Name

4.2. X All geographic subdivisions smaller than a State, including street address, city, county, precinct, zip code, and their equivalent geocodes, except for the initial three digits of a zip code if, according to the current publicly available data from the Bureau of the Census

4.3. X All elements of dates (except year) for dates directly related to an individual, and all ages over 89 and all elements of dates (including year) indicative of such age, except that such ages and elements may be aggregated into a single category of age 90 or older.

4.3.1. X Birth Date ☐ Date of Death

4.3.2. ☐ Discharge date ☐ Admission date

4.3.3. ☐ Appointment Dates ☒ Other Dates (e.g. lab tests, x-rays, MRI, etc.)
HbA1c and LDL blood draw dates, and BP dates

4.4. XTelephone numbers
4.5. ☐ Fax numbers
4.6. ☐ Electronic mail addresses
4.7. X Social Security Number
4.8. ☐ Medical record numbers
4.9. ☐ Health plan beneficiary numbers
4.10. ☐ Account Numbers
4.11. ☐ Certificate/license numbers
4.12. ☐ Vehicle identifiers and serial numbers, including license plate numbers
4.13. ☐ Device identifiers and serial numbers
4.14. ☐ Web universal resource locators (URLS)
4.15. ☐ Internet protocol (IP) address numbers
4.16. ☐ Biometric identifiers, including fingerprints, voiceprints, audio recordings
4.17. ☐ Full-face photographic images and any comparable images
4.18. ☐ Any other unique identifying number, characteristic, or code
4.19. X Personal and Family History
4.20. ☐ History and Physical Examination ☐ Progress Notes
4.21. ☐ Discharge Summary(ies) ☐ Photographs, videotapes, other images
4.22. ☐ X-Ray ☐ HIV (testing or infectious disease) records
4.23. X Diagnostic/Laboratory tests ☐ Sickle cell anemia
4.24. ☐ Drug Abuse Information ☐ Behavioral Health notes
4.25. ☐ Alcoholism or Alcohol Use ☐ Operative Reports
4.26. ☐ Billing records X Medication List
4.27. ☐ Health Summary Reports ☐ Anatomic Pathology Report
4.28. ☒Other Records: Sex and race

5. Will participants be contacted from existing PHI? **X** YES ☐NO ☐N/A
   5.1. If yes, clearly explain how participants will be contacted (NOTE: this would be the same information as listed under section R.8 identification and recruitment of subjects).

Potential participant once identified will first be sent a letter of introduction letting them know about the study and giving them a number if they would like more information or would like for us not to call them. A week after mailing out the letter potential participants will be called and the study will again be introduced to them and they will be asked if they would be interested in participating in the study. See attached letter of contact and script for discussing the project with potential participants on the phone.

6. Provide the titles of the exact individuals who will have access to the collected data.
   6.1. Explain why these individual will have access to this data.

The titles of the individuals with access are Principle Investigator, Project Manager, Program Specialist and Data Base Programmer. They will have access to identify, select, and enroll participants to be part of the study.

W. **Information Security** (*Contact the Information Security Officer for additional assistance regarding confidentiality (storage/security) of research data.)*

1. Provide the precise plan how data is to be collected or acquired (repeat the same information as listed under "Data Collection" section of this form.

Surveys will be read to participants and answers will either be directly input into a computer database or written onto paper forms and then transferred to a database at a later time. The database will reside on VA servers behind the VA firewall and will never be removed from the server. Paper

records will be stored at CHERP behind an electronically locked entrance, a key-locked door and in a key-locked cabinet.

Qualitative interviews will also be read to participants and answers will voice recorded. Interview data will be stored on the VA server behind VA firewalls. Data about the number of calls per month made by the mentor will be collected over the phone, and again responses will either be entered directly into a database or first onto paper and then into the database.

Data about hypoglycemic symptoms will be collected over the phone, and responses will either be entered directly into a database or first onto paper and then onto the database.

Lab results will be obtained from the lab or via CPRS and entered into the secure research database.

2. Provide a listing of the exact research data that will be stored, including but not limited to signed, original informed consent and HIPAA authorization forms, case report forms, etc.

Hba1c, blood pressure, direct LDL, BMI, self-reported demographics, survey data examining diabetes quality of life, depression symptoms, hypoglycemic symptoms, attachment style, goal commitment, diabetes health history, general health history, self-efficacy, self-management behaviors, hypoglycemic symptoms, informed consent form, HIPPA authorization form, and exit interview data.Indicate how project's research data (original and all copies) will be stored and provide corresponding security systems.

Data must be used, stored, and secured according to the requirements of the VHA series 1200 Handbooks, other applicable VA and VHA requirements, and as described in the approved research protocol or as specifically described in the Preparatory to Research Form.

CHERPNAS is a Networked Attached Storage server inside the VA network. It provides space to store electronic data for all CHERP/CEPACT-related studies. It runs Intel Xeon 5600 series 3GHz (64 bit) CPUs with 48GB RAM and 146GB hard drive. Access is limited to the study staff by the database administrator.

Hard copy-based research data will be stored at the CMC VA Medical Center Annex, (file room 17) in a filing cabinet on the second floor. This room is located at CHERP, which is secured by a lock and key, and the filing cabinet containing the data will be secured by lock and key. While files are actively in use, paper records will be held in the CMC VA Medical Center Annex PROMISE (suite 200) in a key-locked filing cabinet behind an electronically locked entrance and a key-locked door. The data will be transported between the CMCVAMC hospital and CMCVAMC Annex in a locked case.

3. Indicate how project's research data (original and all copies) will be stored and provide corresponding security systems. **Project research data both original and copies for hard-copies will be kept on VA property under lock and key in filing cabinets. Electronic data will be kept secure in password protected databases.**

4. CMCVAMC, provide exact location where research data (original and all copies) will be stored and secured.

The CHERP/CEPACT servers are located in the secure CMCVAMC server room, which is the OI&T Server Room that has double locks and a security alarm and cameras, which is located in Room 001 of Building 1 of the main CMCVAMC.

The filing room on the second floor of the Annex and is located at Room 17, and the cabinet is locked inside of Room 17. Active files will be stored in a locked filing cabinet (M133) in PROMISE, Suite 200, located on the second floor of the Annex.

5. Explain how data is to be transported or transmitted from one location to another. **ADA transcription will give us space on their secured server to save our data until it is transcribed then we will erase everything on their server.**
   5.1. Informed Consent discloses PHI transported or transmitted off-site. ☒YES ☐ NO ☐ N/A
   5.2. HIPAA Authorization discloses entities to whom PHI will be transported or transmitted. ☐ YES ☒ NO ☐N/A
      5.2.1. List all entities or individuals outside CMCVAMC to whom data is to be disclosed, and the justification for such disclosure and the authority. ADA Transcription, so the qualitative interviews can be transcribed. We do not have the manpower on staff to transcribe.
   5.3. If yes, list the exact data that will be transmitted. Sept 2015-Dec 2015
   5.4. If yes, explain how data will be protected during transmission outside of CMCVAMC. **This data will be on secured servers with companies that the CMCVAMC previously worked with and approved. In addition, ADA has signed a confidentiality and non-disclosure agreement.**
   5.5. Off-site, provide exact location **ADA Transcription 127 Kings Road Westampton, NJ 08060** (If off-site, attach at least one of the following.)
      5.5.1. Data Use/Transfer Agreement ☒YES ☐NO X N/A
      5.5.2. Off-Site Storage/Transfer of Research Data ☐YES ☐NO **X** N/A
      5.5.3. Memorandum of Understanding ☐YES ☐NO **X** N/A
      5.5.4. *(Note: VA data disclosed to a non-VA investigator at an academic affiliate for research purposes needs to be approved by the Under Secretary of Health or designee.)*

6. List who is to have access to the data and how they are to access it (anyone who has access to the data is responsible for its security).

Judith Long, MD, Anne Canamucio, Jennifer Gutierrez, Tanisha Dicks and Richard Sorrelle, will have full access to the data, through the secure electronic server and the hard copy paper-based data in the locked cabinets. ADA Transcription will have access to the qualitative interviews

7. Describe who is to have access and be responsible for the security of the information (e.g., the Coordinating Center, the statistician, and PI who has ultimate responsibility).

Judith Long who is the Principle Investigator, Anne Canamucio, Jennifer Gutierrez who is the project manager, and Tanisha Dicks and Richard Sorrelle, who are the program specialists, will have full access to the data and be responsible for the security of the information.

8. Provide mechanisms used to account for the information.

The PI will have oversight over all information. Hard copy-based research data will be stored at the CMC VA Medical Center Annex, (file room) in a filing cabinet. This room is located at CHERP, which is secured by a lock and key, and the filing cabinet containing the data will be secured by lock and key. Paper records that are actively in use will be held in the CMC VA Medical Center Annex PROMISE (suite 200) on the second floor, in a key-locked filing cabinet behind an electronically locked entrance and a key-locked door.

CHERPNAS is a Networked Attached Storage server inside the VA network. It provides space to store electronic data for all CHERP/CEPACT-related studies. It runs Intel Xeon 5600 series 3GHz (64 bit) CPUs with 48GB RAM and 146GB hard drive. Access is limited to the study staff by the database administrator.

9. Give security measures that must be in place to protect individually identifiable information if collected or used.

All identifiable information collected by hard copy paper-based data will be kept on VA property under lock and key in the cabinet located in the locked file room. All electronic data will have limited access, granted only to study staff, including the principle investigator and the program specialist under supervision of the database administrator.

10. How and to whom a suspected or confirmed loss of VA information is to be reported.

CMCVAMC Information Security Officer and Privacy Officer will be notified within one hour of the improper use or disclosure, as well as the IRB, Associate Chief of Staff for Research (ACOS/R) and Research Compliance Officer.

11. Identify any circumstances that may warrant special safeguards to protect the rights and welfare of subjects who are likely to be vulnerable including, but not limited to, those subjects who may be susceptible to coercion or undue influence, and describe appropriate actions to provide such safeguards.

Not applicable. We will not be recruiting patients who are considered vulnerable populations or those susceptible to coercion or undue influence.

12. Electronic PHI will be stored on the following:
   - 12.1. CMCVAMC desktop computer with password protection and/or encryption. ☒YES ☐ NO ☐N/A
     - 12.1.1. If yes, identify where the desktop is located. **The CMCVAMC desktops are password protected, but not encrypted. The desktops will be located at CMC VAMC ot the CMC VAMC Annex. Electronic PHI will be stored on VA servers behind the VA fire wall and will not be removed.**
   - 12.2. PVAMC secure server. **X** YES ☐NO ☐N/A
     - 12.2.1. If yes, identify the CMCVAMC server. VISN 4, CMCVAMC/CHERP. CHERPNAS is a Networked Attached Storage server inside the VA network. It provides space to store electronic data for all CHERP/CEPACT-related studies. It runs Intel Xeon 5600 series 3GHz (64 bit) CPUs with 48GB RAM and 146GB hard drive.
     - 12.2.2. External drive that is password protected and/or encrypted. ☐YES **X** NO ☐ N/A
       - 12.2.2.1. If yes, identify the external drive.
   - 12.3. Off-Site server ☐YES **X** NO ☐N/A (<u>If off-site, attach at least one of the following.)</u>
     - 12.3.1. Provide exact location and the name of the <u>off-site</u> server.
     - 12.3.2. Data Use/Transfer Agreement ☐YES ☐NO **X** N/A
     - 12.3.3. Off-Site Storage/Transfer of Research Data ☐YES ☐NO **X** N/A
     - 12.3.4. Memorandum of Understanding ☐YES ☐NO **X** N/A

13. Explain how data is to be transported or transmitted from one location to another. **ADA transcription has secure servers.**

14. Informed Consent discloses PHI transported or transmitted off-site. **X** YES ☐NO ☐N/A

15. HIPAA Authorization discloses entities to whom PHI will be transported or transmitted.
    X YES ☐NO ☐N/A
16. List all entities or individuals outside CMCVAMC to whom data is to be disclosed, and the justification for such disclosure and the authority. Alpha Transcription in order to transcribe the qualitative interviews and the Mixed Methods Research Lab in order to code the interviews

17. Clarify what protection exists for a database. Access is limited to the study staff by the database administrator.

    17.1. Data is stored:
        17.1.1. With identifiers – **X** YES ☐NO
        17.1.2. Coded - ☒YES ☐NO
        17.1.3. De-Identified - ☐YES ☒NO
        17.1.4. Provide the exact list of identifiers that will be stored. Name, social security number, phone number, address, date of birth and age.

18. Describe the plan for protecting research data from improper use or disclosure. Only IRB approved study personnel will have access to study related materials. Study related materials will be kept in password protected files on a secure server. Paper files will be kept on the premises in locked filing cabinets.

    18.1. The Investigator must notify the Information Security Officer, Privacy Officer, IRB, Associate Chief of Staff for Research and Research Compliance Officer within one hour of the improper use or disclosure.

19. Is there a plan to apply for a Certificate of Confidentiality? ☐YES ☐NO ☒N/A
    19.1. If yes, provide a copy of the certificate with this application or to the IRB Office as soon as received.

20. **Record Retention**:
    20.1. The required records, including the investigator's research records, must be retained until disposition instructions are approved by the National Archives and Records Administration and are published in VHA's Records Control Schedule (RCS 10-1). VHA Handbook 1200.05 §26.h
    20.2. Until a schedule for local research records is published, ALL records including identifiers must be retained." ORO/ORD Guidance on Informed Consent Form Modifications Addressing VA Record Retention Requirements (July 23, 2009)

    20.3. If there are additional procedures for record retention, explain further. **None**

X. Qualification of the Investigators

Provide a description of the qualifications of each investigator/co-investigator and their specific role in the study.

Judith A. Long, MD is the primary investigator on this study. She is an experienced researcher in the areas of social determinants of health, health disparities, and Diabetes Mellitus. She is an Associate Professor of Medicine in the Division of General Internal Medicine at the Penn School of Medicine (SOM), the Director of the Penn SOM Masters of Science in Health Policy Research (MSHP), the Principal Investigator (PI) for the Penn NRSA Generalist Research Training Grant, Associate Director of the Penn Robert Wood Johnson Clinical Scholars Program (RWJ CSP) and Co-Chair of the Society for General Internal Medicine (SGIM)

Disparities Task Force. She has more than 10 years of experience in clinical diabetes management. Recently, her research has focused on interventions to reduce disparities in health directing clinical research trials focusing on diabetes. She recently completed a VA based randomized controlled trial (RCT) of peer mentoring and financial incentives for adults with poorly controlled type 2 diabetes and is currently the PI for a similar NIDDK-funded clinical RCT which is being implemented in a non VA low-income minority population. This R01 also includes cost effectiveness analyses similar to those proposed here. She also has a multi-site HSR&D IIR evaluating delivery of care for patients with both diabetes and serious mental illness. She will be responsible for the oversight of this entire study.

1. If applicable, the Principal Investigator must identify a qualified clinician to be responsible for all study related healthcare decisions.

There will be no study related healthcare decisions. Dr. Long will review all serious adverse events and determine if they are potentially study related.

2. PI should submit a current, dated CV with each new initial review.
   Please see attached CV for Judith Long, MD.
