# Supplementary material for: Effect of Peer Mentors in Diabetes Self-management vs Usual Care on Outcomes in US Veterans With Type 2 Diabetes: A Randomized Clinical Trial
Source: JAMA Netw Open. 2020 Sep 11;3(9):e2016369. doi: 10.1001/jamanetworkopen.2020.16369 (PMC7489832; doi:10.1001/jamanetworkopen.2020.16369)
Supplement: Supplement 2. — Data Sharing Statement [file jamanetwopen-e2016369-s002.pdf]

Data Sharing Statement

Long. Effect of Peer Mentors in Diabetes Self-management vs Usual Care on Outcomes in US Veterans With Type 2 Diabetes. *JAMA Netw Open*. Published September 11, 2020.
10.1001/jamanetworkopen.2020.16369

Data
Data available: Yes
Data types: Deidentified participant data, Participant data with identifiers, Data dictionary
How to access data: Please contact Dr. Judith A. Long (jalong@mail.med.upenn.edu) to make a request for data.
When available: With publication

Supporting Documents
Document types: None

Additional Information
Who can access the data: Researchers whose proposed use of the data has been approved.
Types of analyses: Specified purpose.
Mechanisms of data availability: With investigator support, after approval of a proposal, and a signed data access agreement.
